# Contribution of HIF-1α/BNIP3-mediated autophagy to lipid accumulation during irinotecan-induced liver injury

**DOI:** 10.1038/s41598-023-33848-y

**Published:** 2023-04-21

**Authors:** Congjian Shi, Zhenghong Zhang, Renfeng Xu, Yan Zhang, Zhengchao Wang

**Affiliations:** Provincial Key Laboratory for Developmental Biology and Neurosciences, Key Laboratory of Optoelectronic Science and Technology for Medicine of Ministry of Education, College of Life Sciences, Fujian Normal University, No.8, Shangsan Road, Fuzhou, 350007 China

**Keywords:** Molecular medicine, Chaperone-mediated autophagy, Homeostasis

## Abstract

Irinotecan is a topoisomerase I inhibitor which has been widely used to combat several solid tumors, whereas irinotecan therapy can induce liver injury. Liver injury generally leads to tissue hypoxia, and hypoxia-inducible factor-1α (HIF-1α), a pivotal transcription factor, mediates adaptive pathophysiological responses to lower oxygen condition. Previous studies have reported a relationship between HIF-1α and autophagy, and autophagy impairment is a common characteristic in a variety of diseases. Here, irinotecan (50 mg/kg) was employed on mice, and HepG2 and L-02 cells were cultured with irinotecan (10, 20 and 40 μM). In vivo study, we found that irinotecan treatment increased final liver index, serum aminotransferase level and hepatic lipid accumulation. Impaired autophagic flux and activation of HIF-1α/BNIP3 pathway were also demonstrated in the liver of irinotecan-treated mice. Moreover, irinotecan treatment significantly deteriorated hepatic oxidative stress, evidenced by increased MDA and ROS contents, as well as decreased GSH-Px, SOD and CAT contents. Interestingly, protein levels of NLRP3, cleaved-caspase 1 and IL-1β were enhanced in the liver of mice injected with irinotecan. In vitro study, irinotecan-treated HepG2 and L-02 cells also showed impaired autophagic flux, while HIF-1α inhibition efficaciously removed the accumulated autophagosomes induced by irinotecan. Additionally, irinotecan treatment aggravated lipid accumulation in HepG2 and L-02 cells, and HIF-1α inhibition reversed the effect of irinotecan. Furthermore, HIF-1α inhibition weakened irinotecan-induced NLRP3 inflammasome activation in HepG2 cells. Taken together, our results suggest that irinotecan induces liver injury by orchestrating autophagy via HIF-1α/BNIP3 pathway, and HIF-1α inhibition could alleviate irinotecan-induced lipid accumulation in HepG2 and L-02 cells, which will provide a new clue and direction for the prevention of side effects of clinical chemotherapy drugs.

## Introduction

The camptothecin derivative irinotecan is a topoisomerase I inhibitor and has been widely used in the treatment of several solid tumors, including colorectal, pancreatic, ovarian and gastro-esophageal cancers^1^. However, irinotecan therapy can induce liver injury, a portion of patients treated with irinotecan exhibit increased serum liver enzyme levels and develop steatohepatitis^2^. The strong adverse effect can weaken the regenerative capacity of liver remnant and makes patients more susceptible to postoperative liver failure^3,4^, also, colorectal cancer liver metastasis patients with irinotecan-induced injury show an increased risk of morbidity and mortality^5^. Indeed, liver injury may be reversible when chemotherapy is discontinued, whereas in some cases, especially metastatic colorectal cancer and breast cancer, stopping treatment is generally not a sensible option^6^. Currently, there are no effective strategies to prevent and treat liver injury induced by irinotecan, investigating the underlying molecular mechanism may contribute to avoid redundant pathological events and to optimize the efficacy.

Liver injury may lead to tissue hypoxia through changing the liver perfusion or mitochondrial modification, and emerging evidences demonstrate that hypoxia promotes hepatic lipid accumulation, inflammation and fibrosis via activating hypoxia-inducible factors (HIFs) and their downstream targets^7–10^. HIFs are heterodimeric complexes consisting of the oxygen-sensitive α subunit and the constitutively expressed β subunit, there are three subunits of HIF-α: HIF-1α, HIF-2α and HIF-3α, of which HIF-1α is the best described HIFs and expressed in almost all cells^11^. Derangements in HIF-1α pathway have been reported during the development of alcoholic and non-alcoholic fatty liver disease^12,13^.

Autophagy is a lysosome-dependent, self-degradative process that plays fundamental roles during coping with a series of cellular stress, including starvation, hypoxia and damaged organelles^14,15^. Lipid droplets can be regarded as autophagic substrates for degradation^16^ and recent studies proposed that activation of autophagy leads to alleviated non-alcoholic fatty liver disease^17^. The tolerable level of autophagy plays indispensable roles in suppressing oxidative stress, lipid accumulation, protein aggregation, inflammation and chronic cell death, thus the modulation of autophagy distinctly affects the development of liver injury^18^. Notably, HIF-1α is a significant regulator of autophagy by cooperating with its downstream target, BNIP3^10,19^.

In the current study, we demonstrated the activation of HIF-1α/BNIP3 pathway and impaired autophagic flux in the liver of irinotecan-treated mice and in irinotecan-treated HepG2 and L-02 cells, also, irinotecan treatment intensified oxidative stress and NLRP3 inflammasome activity. More importantly, inhibition of HIF-1α attenuated irinotecan-induced autophagosome accumulation, lipid deposition and NLRP3 inflammasome activation in cultured cells, which will provide a new clue and direction for the prevention of side effects of clinical chemotherapy drugs.

## Materials and methods

### Animals and experiments design

Male C57BL/6 mice (8-week-old) were supplied by the Experimental Animal Center of Fujian Normal University for irinotecan-induced liver injury model. The study was carried out in compliance with the ARRIVE guidelines. All experiments were conducted in accordance with relevant guidelines and regulations and all animal testing procedures were approved by the Animal Care and Use Committee of Fujian Normal University. The animals were housed in specialized plastic cages with continuous supply of food and water, and maintained at standard conditions (23 ± 2 °C, 12 h/12 h light/dark schedule). After the adaption for 2 weeks, mice were randomly divided into normal control (NC, n = 8) and irinotecan (IR, n = 8) groups. IR group was administered 50 mg/kg irinotecan (GlpBio, GC11473) and NC group was administered equal volumes of solvent (saline) via intraperitoneal injection every three days for 3 weeks. The dosage of irinotecan is based on previous studies^20,21^. On day 22, 9 h after irinotecan injection, mice were killed by CO_2_ inhalation and blood samples were collected for further analyses. The livers were also removed and weighed rapidly, all liver tissues were fixed in 4% paraformaldehyde, snap-frozen in OCT or stored at − 80 °C.

### Serum aminotransferase measurements

The activities of serum alanine aminotransferase (ALT) and aspartate aminotransferase (AST) were detected using commercial assay kits (Jiancheng, Nanjing, China) with a microplate reader (Biotek, USA).

### Hepatic biochemical analysis

After homogenization with cold PBS, triglyceride (TG), malondialdehyde (MDA), glutathione peroxidase (GSH-Px), superoxide dismutase (SOD) and catalase (CAT) in liver tissues were quantified using commercial assay kits (Jiancheng, Nanjing, China) according to the manufacturer’s protocols.

### Histological analysis

4% paraformaldehyde-fixed liver tissues were embedded in paraffin and 5 μm thick sections were stained with hematoxylin and eosin. Frozen sections were cut from liver tissues snap-frozen in OCT and mounted on glass slides for Oil Red O staining.

### Immunofluorescence analysis

Frozen liver tissues were sectioned and fixed in 10% neutral formalin. Then, the liver sections were incubated with indicated primary antibody overnight at 4 °C. On the next day, the sections were incubated with the corresponding secondary antibody for 60 min at room temperature. Nuclei were stained with 4′,6-diamidino-2-phenylindole (DAPI) (Beyotime, China) for 5 min at room temperature. Hypoxic regions were detected using pimonidazole (MCE, HY-105129A) as described previously^22^. Images were obtained by a fluorescence microscope (Olympus, Tokyo, Japan).

### Acridine orange (AO) staining

After deparaffinization and hydration, the paraffin sections of liver were stained with AO (Sangon Biotech, Shanghai, China) in darkness at room temperature for 30 min. Subsequently, images were obtained by a fluorescence microscope (Olympus, Tokyo, Japan).

### Reactive oxygen species (ROS) detection

The effect of irinotecan on ROS level in liver tissues was evaluated using a DHE-ROS assay kit (BestBio, Shanghai, China). DHE can pass through cell membrane and be oxidized by ROS to generate a novel product that incorporates into DNA, thus producing intracellular red fluorescence^23^. Briefly, liver tissues were incubated with DHE in darkness at 37 °C for 30 min, the fluorescence was detected by a fluorescence microscope (Olympus, Tokyo, Japan).

For intracellular detection, cells were washed three times with PBS and incubated with fluorescence probe DCFH-DA (Beyotime, China) at 37 °C for 30 min. The fluorescence of 2 × 10^4^ cells was detected by a microplate reader (Biotek, USA) at excitation and emission wavelengths of 488 and 525 nm, respectively.

### Cell culture and treatment

HepG2 and L-02 cells were purchased from the Cell Bank of the Chinese Academy of Sciences (Shanghai, China) and maintained in a humidified atmosphere of 5% CO2 at 37 ℃. DMEM (Hyclone, USA) containing 10% fetal bovine serum (FBS, Gibco, USA), 1% penicillin and streptomycin was used for cell culture. Cells were stimulated for 12 h with irinotecan (0, 10, 20 and 40 μM) and echinomycin (6 nM) (GlpBio, GC18236) was administrated during the last 6 h of 12 h-treatment.

### CCK8 assay

HepG2 cells were seeded in 96-well plates at a density of 5 × 10^3^ cells in 100 μL medium each well. Then, the cells were exposed to different concentrations of irinotecan (0, 1, 5, 10, 20, 40, 80 and 160 μM) for 12 h, and the CCK8 assay (Beyotime, China) was used to measure cell viability according to the manufacturer’s protocol.

### HIF-1α DNA binding activity

HIF-1α DNA binding activity was measured using a commercial assay kit (Abcam, ab133104). Briefly, nucleoprotein was extracted from HepG2 cells using a Nuclear Extraction Kit (Beyotime, China). Then, the obtained samples were added to the wells of transcription factor HIF-1α plate and incubated overnight at 4 °C. After incubation, diluted HIF-1α primary antibody was added to each well and incubated for 60 min at room temperature. Subsequently, diluted goat anti-rabbit HRP conjugate was added to each well and incubated for 60 min at room temperature. HIF-1α DNA binding activity were measured at 450 nm using a microplate reader (Biotek, USA).

### Small interfering RNA (siRNA) transfection

HepG2 cells were transfected with siRNA targeting HIF-1α (GenePharma, Shanghai, China) or scrambled siRNA according to the instructions of lipofectamine™ 2000 (Invitrogen, Carlsbad, CA, USA). siRNA targeting HIF-1α with the antisense strand: 5′-AUC AAG AUG CGA ACU CAC ATT-3′ and scrambled siRNA with the antisense strand: 5′-GAC UAC UGG UCG UUG ATT-3′. 6 h after transfection, the culture medium was changed and irinotecan was added.

### Intracellular TG measurement

Lipid droplets can be quantified by TG content. Cells were stimulated with designated reagents and TG content was measured using a commercial assay kit (Jiancheng, Nanjing, China) according to the manufacturer’s protocol.

### Western blot analysis

Liver tissues and cultured cells were lysed in ice-cold radioimmunoprecipitation assay (RIPA) buffer to obtain extract and protein concentration was measured using a bicinchoninic acid (BCA) protein assay kit (Beyotime, China). Protein samples were electrophoresed in sodium dodecyl sulfate–polyacrylamide gel electrophoresis (SDS-PAGE) and transferred to polyvinylidene difluoride (PVDF) membranes (Millipore, USA). After blocking with 5% skim milk for 2 h, the membranes were placed overnight at 4 °C with indicated primary antibodies (Supplementary Table S1). The membranes were cut prior to hybridisation with indicated primary antibodies. The membranes were then incubated with appropriate secondary horseradish peroxidase-conjugated IgG antibodies (Beyotime, China). Protein bands were visualized using an enhanced chemiluminescence (ECL) kit (Beyotime, China) and quantitatively analyzed with Image J Software.

### Statistical analysis

All experimental data were analyzed using GraphPad Prism 7.0 (San Diego, CA, USA)^17^ and SPSS 22.0 (Chicago, IL, USA)^24^. The results were expressed as the mean ± SD. Statistical comparisons between groups were made by one-way analysis of variance (ANOVA) followed by Duncan’s test. Differences were considered statistically significant if the *P* value was less than 0.05.

## Results

### Irinotecan induces liver injury in mice

**Figure 1:**
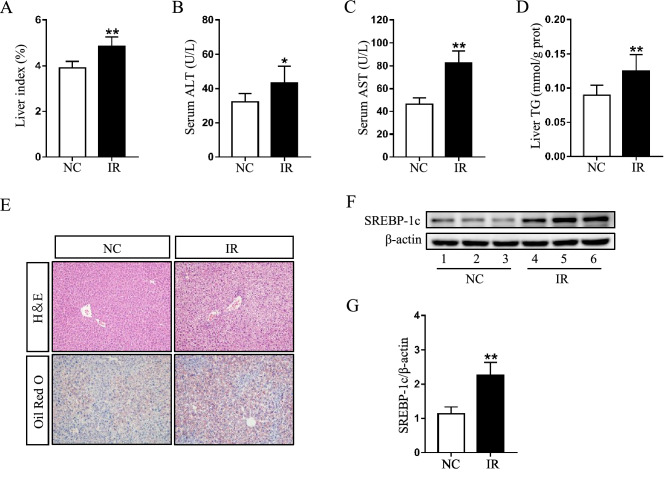
Irinotecan (IR) induces liver injury in mice. Mice were intraperitoneally injected with IR (50 mg/kg) or solvent (saline) every three days for 3 weeks. Effects of IR on (**A**) liver index, serum levels of (**B**) ALT and (**C**) AST, and (**D**) hepatic TG content. Data are presented as mean ± SD (n = 8), *P < 0.05 and **P < 0.01 compared with normal control (NC) group. (**E**) Liver sections were stained with H&E and Oil Red O to evaluate lipid accumulation. (**F**) Western blot analysis of SREBP-1c protein level in liver tissue. (**G**) Densitometric quantification of SREBP-1c. The blots were cut prior to hybridisation with indicated primary antibodies. Data are presented as mean ± SD (n = 6), **P < 0.01 compared with NC group.

All of mice injected with irinotecan were characterized by evident liver injury. Firstly, the final liver index in IR group was significantly higher than that in NC group (Fig. 1A), revealing that irinotecan can augment the swelling of liver. Further, irinotecan-treated mice showed elevated serum ALT (Fig. 1B) and AST activity (Fig. 1C). Liver index, ALT and AST were regarded as indicators of liver injury. Level of hepatic TG was also significantly increased in IR group as compared to NC group (Fig. 1D). For histopathological analysis, HE and Oil Red O staining were performed on liver sections. As shown in Fig. 1E, the administration of irinotecan induced intrahepatic microvesicular steatosis, ballooning degeneration and portal neutrophil infiltration (HE staining), meanwhile, liver tissues in irinotecan-treated mice exhibited aggravated lipid accumulation (Oil Red O staining). SREBP-1c is capable of activating genes that involve in triglyceride and fatty acid synthesis, we then observed a significant increase of SREBP-1c protein level in liver tissues from IR group when compared with NC group (Fig. 1F,G).

### Impaired autophagic flux and activated HIF-1α/BNIP3 pathway in irinotecan-induced liver injury

**Figure 2:**
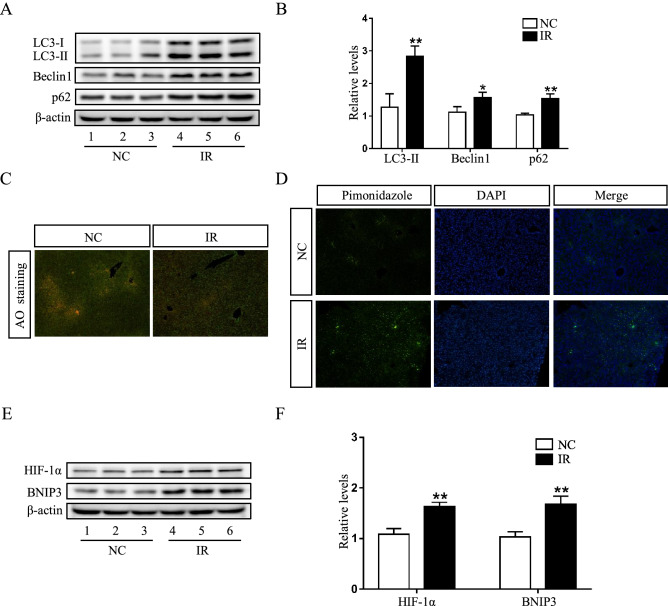
Impaired autophagic flux and activated HIF-1α/BNIP3 pathway are demonstrated in the liver of irinotecan (IR)-treated mice. (**A**) Western blot analysis of LC3-II, Beclin1 and p62 protein levels in liver tissue. (**B**) Densitometric quantification of LC3-II, Beclin1 and p62. (**C**) Effects of irinotecan on the formation of hepatic acidic vesicular organelles (orange) was examined by acridine orange (AO) staining. (**D**) Immunofluorescence was performed on liver sections using pimonidazole (green) to detect hypoxic regions, nuclei were stained with DAPI (blue). (**E**) Western blot analysis of HIF-1α and BNIP3 protein levels in liver tissue. (**F**) Densitometric quantification of HIF-1α and BNIP3. The blots were cut prior to hybridisation with indicated primary antibodies. Data are presented as mean ± SD (n = 6), *P < 0.05 and **P < 0.01 compared with normal control (NC) group.

Growing evidence has shown that autophagy plays a role in the pathogenesis of liver diseases, including viral hepatitis, non-alcoholic fatty liver disease and hepatocellular carcinoma^25,26^, we hypothesized that irinotecan-induced liver injury could be related with the autophagic pathway. Subsequent experiment results showed that the protein levels of LC-3II, Beclin1 and p62 was significantly upregulated in the liver of IR group as compared to NC group (Fig. 2A,B). Beclin1 is an established autophagy inducer, LC-3II firmly attaches to autophagosome membrane and the level of LC-3II is linked to the quantity of autophagosome, whereas p62 can be degraded by autophagic process and accumulates once autophagy is impaired^27,28^. The highly acidic condition in lysosome is conducive to autophagic process, in the present study, we used acridine orange, which can pass through cell membrane and accumulate in acidic vesicular organelles, to assess lysosomal function, and liver tissues in irinotecan-treated mice showed a decreased fluorescence intensity (Fig. 2C). These results indicated an impediment in autophagosome degradation during the development of irinotecan-induced liver injury.

Since liver injury generally leads to tissue hypoxia and HIF-1α is capable of mediating adaptive physiological and pathological responses to lower oxygen condition. Expectedly, the hypoxic regions stained with hypoxia marker pimonidazole was obviously increased in response to irinotecan treatment (Fig. 2D). Also, the protein level of HIF-1α was significantly upregulated in IR group as compared to NC group (Fig. 2E,F), and the protein level of HIF-1α downstream target, BNIP3 was also concomitant upregulated (Fig. 2E,F). These data revealed that HIF-1α/BNIP3 pathway was activated during the liver injury induced by irinotecan.

### Effects of irinotecan on hepatic oxidative stress and NLRP3 inflammasome activity

**Figure 3:**
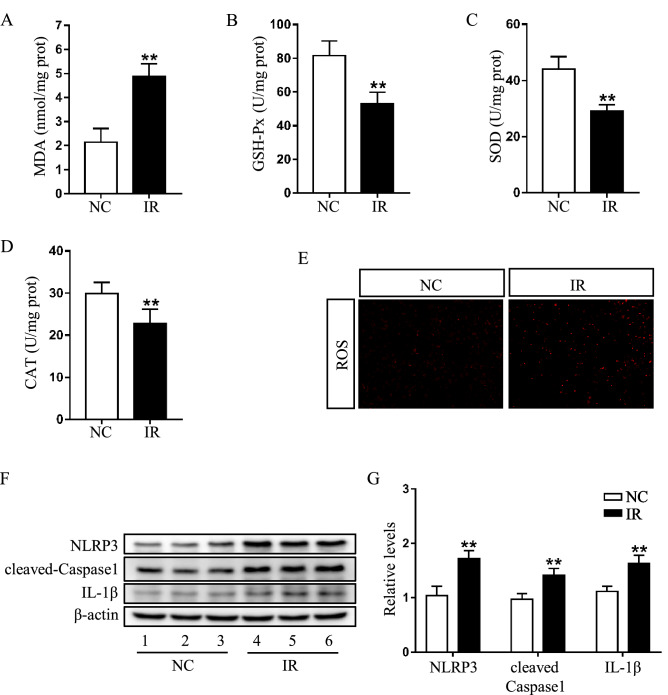
Irinotecan (IR) aggrandizes hepatic oxidative stress and NLRP3 inflammasome activity in mice. Effects of IR on hepatic (**A**) MDA, (**B**) GSH-Px, (**C**) SOD and (**D**) CAT content. Data are presented as mean ± SD (n = 8), **P < 0.01 compared with normal control (NC) group. (**E**) Hepatic ROS level were determined by DHE staining. Red color is considered as ROS staining. (**F**) Western blot analysis of NLRP3, cleaved-Caspase1 and IL-1β protein levels in liver tissue. (**G**) Densitometric quantification of NLRP3, cleaved-Caspase1 and IL-1β. The blots were cut prior to hybridisation with indicated primary antibodies. Data are presented as mean ± SD (n = 6), **P < 0.01 compared with NC group.

HIF-1α has been involved in the regulation of ROS during hypoxia^29^. In our study, the biochemical analysis indicated that MDA (Fig. 3A) level was higher, while GSH-Px (Fig. 3B), SOD (Fig. 3C) and CAT (Fig. 3D) levels were lower in the liver of IR group as compared to NC group. Additionally, a marked increase of DHE-positive staining was confirmed in the liver of IR group (Fig. 3E). These results demonstrated that irinotecan aggravated hepatic oxidative stress in mice.

Previous studies reported that autophagy impairment was related with excessive activation of inflammasome and ROS was a known regulator of NLRP3 inflammasome^30,31^. We then measured the activity of NLRP3 inflammasome in liver tissues, based on the results of western blot, we found that the protein levels of NLRP3, cleaved-caspase1 and IL-1β were significantly upregulated in IR group (Fig. 3F,G). These data suggested that irinotecan caused a dysregulated activation of NLRP3 inflammasome in irinotecan-induced liver injury.

### Inhibition of HIF-1α pathway mitigates autophagosome accumulation and lipid deposition in irinotecan-treated HepG2 and L-02 cells

**Figure 4:**
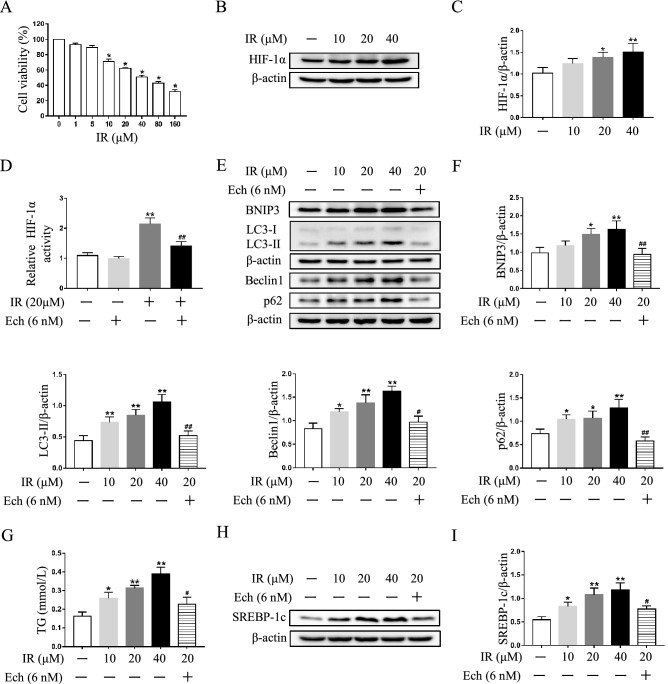
Echinomycin (Ech) mitigates autophagosome accumulation and lipid deposition in irinotecan (IR)-treated HepG2 cells. HepG2 cells were exposed to media supplemented with IR (10, 20, 40 μM) for 12 h-treatment and Ech (6 nM) were given during the last 6 h of 12 h-treatment. (**A**) Cell viability of HepG2 cells treated with different concentrations of irinotecan is measured by CCK8 assay. Data are presented as mean ± SD (n = 6), *P < 0.05 compared with 0 μM group. (**B**) Western blot analysis of HIF-1α protein level in HepG2 cells. (C) Densitometric quantification of HIF-1α. (**D**) Relative HIF-1α DNA binding activity in each group. (**E**) Western blot analysis of BNIP3, LC3-II, Beclin1 and p62 protein levels in HepG2 cells. (**F**) Densitometric quantification of BNIP3, LC3-II, Beclin1 and p62. (**G**) IR exacerbated TG content in HepG2 cells and co-treated with Ech decreased cellular TG content. (**H**) Western blot analysis of SREBP-1c protein level in HepG2 cells. (**I**) Densitometric quantification of SREBP-1c. The blots were cut prior to hybridisation with indicated primary antibodies. Data are presented as mean ± SD (n = 3), *P < 0.05 and **P < 0.01 compared with normal control group, ^#^*P* < 0.05 and ^##^*P* < 0.01 compared with 20 μM IR-treated group.

**Figure 5:**
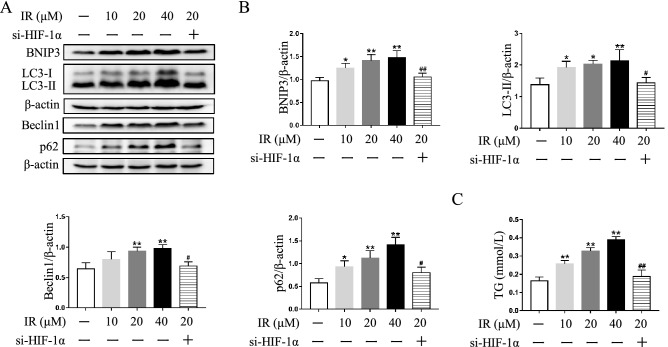
HIF-1α siRNA mitigates autophagosome accumulation and lipid deposition in irinotecan (IR)-treated HepG2 cells. (**A**) Western blot analysis of BNIP3, LC3-II, Beclin1 and p62 protein levels in HepG2 cells. (**B**) Densitometric quantification of BNIP3, LC3-II Beclin1 and p62. (**C**) IR exacerbated TG content in HepG2 cells and co-treated with HIF-1α siRNA decreased cellular TG content. The blots were cut prior to hybridisation with indicated primary antibodies. Data are presented as mean ± SD (n = 3), *P < 0.05 and **P < 0.01 compared with normal control group, ^#^*P* < 0.05 and ^##^*P* < 0.01 compared with 20 μM IR-treated group.

**Figure 6:**
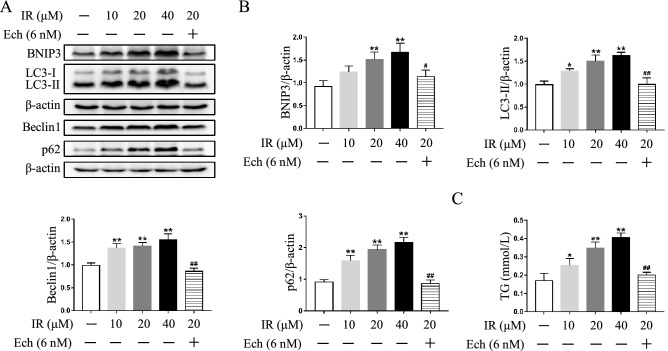
Echinomycin (Ech) mitigates autophagosome accumulation and lipid deposition in irinotecan (IR)-treated L-02 cells. L-02 cells were exposed to media supplemented with IR (10, 20, 40 μM) for 12 h-treatment and Ech (6 nM) were given during the last 6 h of 12 h-treatment. (**A**) Western blot analysis of BNIP3, LC3-II, Beclin1 and p62 protein levels in L-02 cells. (**B**) Densitometric quantification of BNIP3, LC3-II, Beclin1 and p62. (**C**) IR exacerbated TG content in L-02 cells and co-treated with Ech decreased cellular TG content. The blots were cut prior to hybridisation with indicated primary antibodies. Data are presented as mean ± SD (n = 3), *P < 0.05 and **P < 0.01 compared with normal control group, ^#^*P* < 0.05 and ^##^*P* < 0.01 compared with 20 μM IR-treated group.

To verify the involvement of HIF-1α pathway in autophagic process under irinotecan treatment, we used echinomycin or HIF-1α siRNA to inhibit HIF-1α activity and then detected protein levels of HIF-1α downstream target and autophagy markers in cultured cells. Initially, cell viability of HepG2 cells treated with different concentrations of irinotecan was measured, the data showed significant differences from 10 to 160 μM irinotecan group when compared with the control group (Fig. 4A). Thus, 10 μM, 20 μM and 40 μM irinotecan were select for subsequent research, and the protein level of HIF-1α was markedly increased by irinotecan treatment in a dose-dependent manner in HepG2 cells (Fig. 4B,C). We also identified the inhibitory effect of echinomycin on HIF-1α, and the result indicated that echinomycin obviously decreased HIF-1α DNA binding activity in HepG2 cells under irinotecan treatment (Fig. 4D). Then, the results in Fig. 4E,F showed that echinomycin treatment markedly compromised the protein level of BNIP3 and consistently decreased the protein levels of LC-3II and Beclin1 in irinotecan-treated HepG2 cells. We also observed the alleviation of p62 accumulation in irinotecan-treated HepG2 cells after inhibiting HIF-1α pathway. Next, we checked the effect of HIF-1α knockdown in irinotecan-treated HepG2 cells, HIF-1α siRNA significantly inhibited the protein level of HIF-1α in HepG2 cells under irinotecan treatment (Supplementary Fig. S1), and the protein levels of BNIP3, LC-3II, Beclin1 and p62 were consistent with echinomycin administration (Fig. 5A,B). Considering that irinotecan is an anticancer drug, we also used echinomycin to inhibit HIF-1α in irinotecan-treated L-02 cells for further verification, and western blot results were consistent with those in HepG2 cells (Fig. 6A,B). Overall, the above results demonstrated that HIF-1α pathway was involved in autophagic process and inhibition of HIF-1α pathway mitigates autophagosome accumulation in irinotecan-treated HepG2 and L-02 cells.

Consistent with in vivo study, irinotecan treatment obviously increased cellular TG level while co-treatment with echinomycin suppressed the effect of irinotecan in HepG2 cells (Fig. 4G). Meanwhile, western blot result showed higher expression of SREBP-1c in irinotecan-induced HepG2 cells as compared to the control group, and echinomycin treatment greatly decreased the expression of SREBP-1c in irinotecan-induced HepG2 cells (Fig. 4H,I). The increase of intracellular TG level caused by irinotecan could also be abolished by si-HIF-1α in HepG2 cells (Fig. 5C). Likewise, inhibition of HIF-1α by echinomycin weakened irinotecan-induced TG level increase in L-02 cells (Fig. 6C). Taken together, these results revealed that HIF-1α inhibition ameliorated lipid deposition in irinotecan-treated HepG2 and L-02 cells.

### Increased NLRP3 inflammasome activity is mediated by HIF-1α pathway in irinotecan-treated HepG2 cells

**Figure 7:**
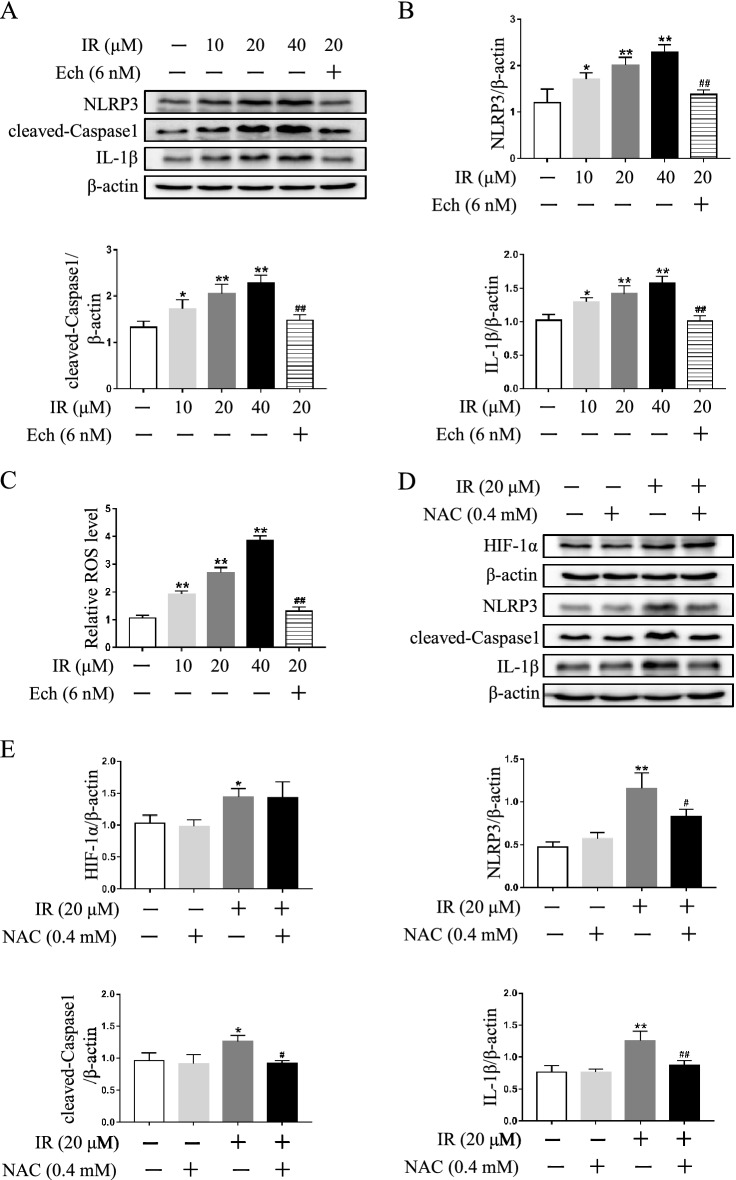
HIF-1α is involved in the activation of NLRP3 inflammasome in irinotecan (IR)-treated HepG2 cells. (**A**) Echinomycin (Ech) treatment suppresses NLRP3 inflammasome activity in IR-treated HepG2 cells. Western blot analysis of NLRP3, cleaved-Caspase1 and IL-1β protein levels in HepG2 cells. (**B**) Densitometric quantification of NLRP3, cleaved-Caspase1 and IL-1β. (**C**) Ech treatment alleviates ROS production in IR-treated HepG2 cells. Relative ROS level in each group. (**D**) Effects of ROS production on HIF-1α and NLRP3 inflammasome activity in IR-treated HepG2 cells. Western blot analysis of HIF-1α, NLRP3, cleaved-Caspase1 and IL-1β protein levels in HepG2 cells. (**E**) Densitometric quantification of HIF-1α, NLRP3, cleaved-Caspase1 and IL-1β. The blots were cut prior to hybridization with indicated primary antibodies. Data are presented as mean ± SD (n = 3), *P < 0.05 and **P < 0.01 compared with normal control group, ^#^*P* < 0.05 and ^##^*P* < 0.01 compared with 20 μM IR-treated group.

Next, the effect of irinotecan on NLRP3 inflammasome activity was investigated in HepG2 cells, and an increase was detected under irinotecan treatment, while inhibition of HIF-1α by echinomycin significantly suppressed NLRP3 inflammasome activity induced by irinotecan (Fig. 7A,B). Meanwhile, the tendency of ROS level was consistent with NLRP3 inflammasome activity (Fig. 7C), implying that ROS may be the pivotal factor in activating NLRP3 inflammasome during this pathological process. More importantly, preincubation with the ROS scavenger N-acetylcysteine (NAC) tellingly decreased NLRP3 inflammasome activity in irinotecan-treated HepG2 cells, but had no obvious effect on the protein level of HIF-1α (Fig. 7D,E). These results suggested that aberrant NLRP3 inflammasome activation was mediated by HIF-1α pathway and played a significant role in irinotecan-treated HepG2 cells.

## Discussion

Our present study clearly demonstrated that irinotecan induces liver injury by orchestrating autophagy via HIF-1α/BNIP3 pathway, and HIF-1α inhibition could alleviate irinotecan-induced lipid accumulation in HepG2 and L-02 cells, indicating the contribution of HIF-1α/BNIP3-mediated autophagy to lipid accumulation during irinotecan-induced liver injury. This finding will provide a new clue and direction for the prevention of side effects of clinical chemotherapy drugs.

Irinotecan, alone or in combination, has been widely used in the treatment of several solid tumors and exerts strong antitumor activity by specifically inhibiting topoisomerase I^32^. Despite impressive efficacy and improved patient survival, growing evidence suggests that irinotecan therapy is linked to the development of liver injury^2,33,34^. Irinotecan-containing regimens have deleterious effects on liver parenchyma and lead to impaired liver regeneration, thus patients with liver injury induced by irinotecan may develop steatohepatitis, cirrhosis or liver failure in the long term^5,6^. Furthermore, such hepatic lesions limit metastasectomy and observably increase the risk of morbidity and mortality in colorectal cancer liver metastasis patients^35^. Irinotecan-induced liver injury is related with the disruption of lipid homeostasis and inflammation, however, the underlying mechanisms are not well understood. In the present study, we initially observed an increase of final liver index in irinotecan-treated mice, liver index is normally linked to the extent of liver swelling^36^. Hepatocellular ALT and AST can be released into blood in response to cellular damage^37^, and irinotecan markedly elevated the levels of serum ALT and AST. Liver index, ALT and AST have long been regarded as the indicators of liver injury. In addition, irinotecan resulted in the disruption of hepatic lipid homeostasis, as evidenced by superfluous hepatic TG content and increased SREBP-1c expression. The above results showed that irinotecan induced liver injury in mice, and this was further confirmed by histopathological analysis.

Autophagy is a lysosome-dependent, self-degradative process that plays vital roles in cell maintenance and survival, and dysregulation of autophagy is verified in a large variety of human diseases^38^. Particularly, autophagy represents an inducible response to stimulation including oxidative stress, lipid accumulation, protein aggregate, inflammation and chronic cells death^18^. It has been reported that inhibition of autophagy by autophagy-related gene 7 (Atg7) conditional knockout in mice eventually leads to liver injury with hepatomegaly^39^. Autophagy also plays essential roles in maintaining hepatic lipid homeostasis, while lipid droplets can be sequestrated by autophagosomes, subsequently leading to the degradation through autophagic pathway, so the loss of autophagy has been shown to facilitate lipid accumulation in both cultured hepatocytes and mouse livers^40^. Our present results demonstrated that the expression of LC-3II was concomitantly increased with the upregulation of Beclin1 in the liver of irinotecan-treated mice. Beclin1 is an established autophagy inducer, while LC-3II is membrane bound and can serve as a specific marker of autophagosome^41^. The elevated expression of LC-3II does not necessarily indicate the active autophagy, as accumulated autophagosomes may represent an impediment of degradation process^42^. Next, we observed elevated expression of p62, a protein that indicates autophagosome degradation, in the liver of irinotecan-treated mice. Furthermore, Nguyen et al. indicated that SREBP-1c activation was associated with impaired autophagic flux in diet-induced non-alcoholic fatty liver disease model^43^. Acridine orange staining also suggested decreased lysosome function in the liver of irinotecan-treated mice. Taken together, the results of p62, SREBP-1c and acridine orange staining in our study have showed that autophagic flux was impaired in irinotecan-induced liver injury.

Given that liver injury can induce tissue hypoxia^44^, we measured the expression change of HIF-1α and then observed increased HIF-1α expression in the liver of irinotecan-treated mice. Moreover, we detected the expression of BNIP3, a downstream target of HIF-1α that is involved in autophagy process, and the result showed that the expression of hepatic BNIP3 was significantly increased by irinotecan injection. BNIP3 can replace Beclin1 in Bcl-2-Beclin1 or Bcl-XL-Beclin1 complexes, and dissociated Beclin1 promotes autophagosome formation^45^. These data testified that HIF-1α/BNIP3 pathway was activated in the liver of irinotecan-treated mice. Further, HIF-1α has been involved in the regulation of ROS during hypoxia^29^, our data clearly demonstrated that increased MDA and ROS levels were accompanied by decreased levels of GSH-Px, SOD and CAT in the liver of irinotecan-treated mice. MDA is identified as the marker of oxidative injury, whereas GSH-Px, SOD and CAT are crucial antioxidant agents that can efficaciously eliminate ROS^46^. Consistent with our findings, previous reports also demonstrated the aggravated oxidative stress in irinotecan-induced liver injury^5,35^.

Inflammasomes are multimeric protein complexes that carry out inflammatory cascades in response to pathogens and endogenous dangers^47^. NLRP3 inflammasome is the most thoroughly investigated inflammasome complex and a wide range of stimuli are capable of triggering its activation, including ROS, potassium outflow, calcium influx, mitochondrial dysfunction and endoplasmic reticulum stress^48^. Interestingly, we also found increased NLRP3 inflammasome activity in the liver of irinotecan-treated mice, implying that aberrant NLRP3 inflammasome activation may also play a role during this pathological process.

Further analysis of in vitro cell experiments clearly demonstrated that irinotecan also caused the impairment of autophagic flux in HepG2 and L-02 cells, and inhibition of HIF-1α decreased irinotecan-induced BNIP3, LC-3II and Beclin1 expressions, and curtailed the accumulation of autophagosome which had a salutary effect on impaired autophagic flux. These results indicated that HIF-1α/BNIP3 pathway was involved in autophagic process, and inhibition of HIF-1α mitigated autophagosome accumulation in irinotecan-treated HepG2 and L-02 cells. In addition, irinotecan exacerbated lipid accumulation in HepG2 and L-02 cells, notably, inhibition of HIF-1α suppressed the effect of irinotecan which may be related to the clearance of accumulated autophagosomes, this finding is consistent with another study verifying that inhibition of HIF-1α by small interfering RNA restrains monocyte chemoattractant protein-1 (MCP-1)-induced lipid accumulation in Huh7 cells^49^. Previous investigations have found that the activity of NLRP3 inflammasome can be regulated by HIF-1α^50,51^, consistently, our results also showed that aberrant NLRP3 inflammasome activation was mediated by HIF-1α pathway and played a significant role in irinotecan-treated HepG2 cells.

In conclusion, our present study clearly revealed that irinotecan induces liver injury by modulating autophagy via HIF-1α/BNIP3 pathway, and the strategies targeting HIF-1α signaling may have a protective effect on liver injury induced by irinotecan.

## Supplementary Information

Supplementary Information 1.

Supplementary Information 2.

## Data Availability

The original contributions presented in the study are included in the article, and further inquiries can be directed to the corresponding author.
