# Supplementary material for: Contribution of HIF-1α/BNIP3-mediated autophagy to lipid accumulation during irinotecan-induced liver injury
Source: Sci Rep. 2023 Apr 21;13:6528. doi: 10.1038/s41598-023-33848-y (PMC10121580; doi:10.1038/s41598-023-33848-y)
Supplement: Supplementary file 1 — Supplementary Information 1. [file 41598_2023_33848_MOESM1_ESM.pdf]

Figure S1:

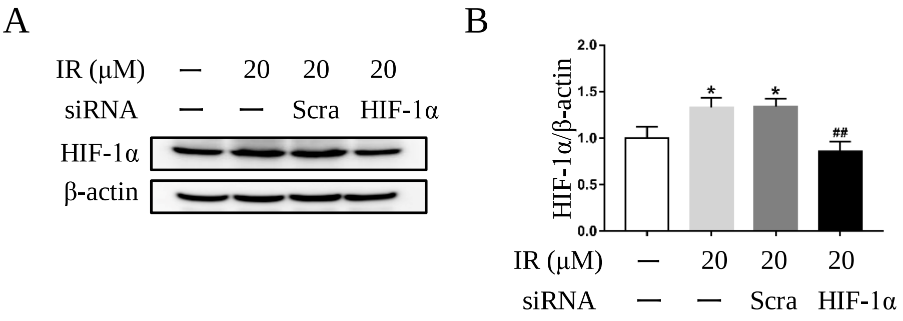

Figure S1. HIF-1α siRNA decreases the protein level of HIF-1α in irinotecan (IR)-treat HepG2 cells. (A) Western blot analysis of HIF-1α protein level in HepG2 cells. (B) Densitometric quantification of HIF-1α. The blots were cut prior to hybridisation with indicated primary antibodies. Data are presented as mean ± SD (n = 3), *P < 0.05 compared with normal control group, ##P < 0.01 compared with Scra group. Scra: scrambled.

Table S1:

Table S1. Antibody information for western blot.

**Antibody Name**	**Company and City**	**Dilution Degree**
HIF-1α	Gene Tex, Irvine, CA, USA	1:2000
NLRP3	Affinity Biosciences, Cincinnati, OH, USA	1:1000
LC-3I/II	Abcam, Cambridge, MA, USA	1:1000
p62	Abcam, Cambridge, MA, USA	1:1000
BNIP3	Bioss, Beijing, China	1:1000
SREBP-1c	Bioss, Beijing, China	1:1000
IL-1β	Protein Tech Group, Wuhan, China	1:1000
Beclin1	Protein Tech Group, Wuhan, China	1:5000
cleaved Caspase-1	Protein Tech Group, Wuhan, China	1:1000
β-actin	Protein Tech Group, Wuhan, China	1:5000
anti-Mouse IgG	Beyotime, Haimen, China	1:1000
anti-Rabbit IgG	Beyotime, Haimen, China	1:1000
