# Supplementary material for: Contribution of HIF-1α/BNIP3-mediated autophagy to lipid accumulation during irinotecan-induced liver injury
Source: Sci Rep. 2023 Apr 21;13:6528. doi: 10.1038/s41598-023-33848-y (PMC10121580; doi:10.1038/s41598-023-33848-y)

Figure 1F:

SREBP-1c

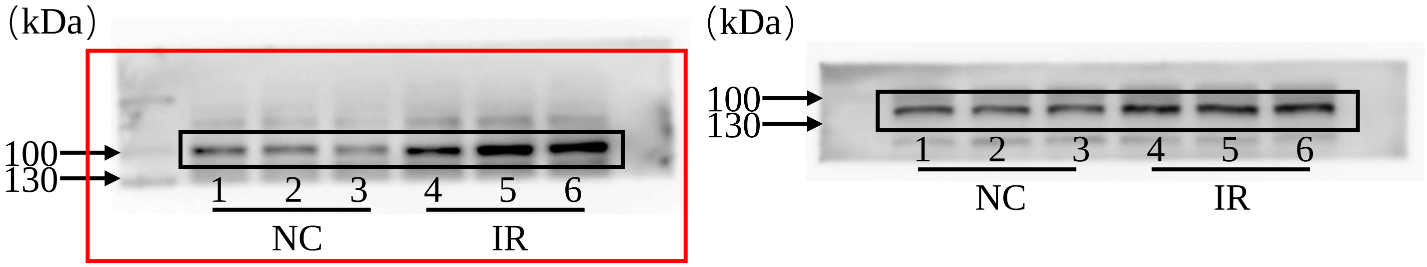

β-actin

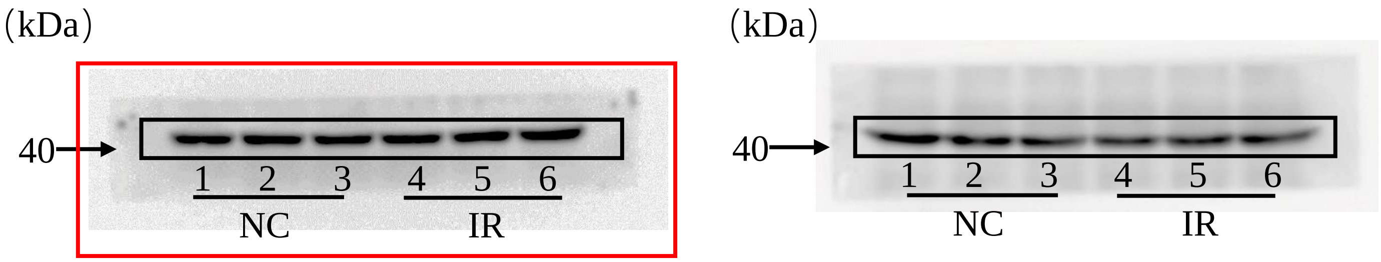

Figure 2A:

LC3

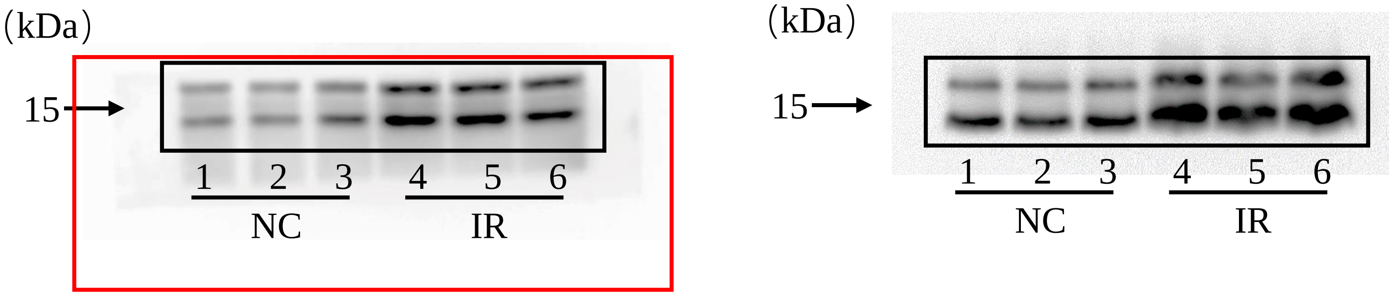

Beclin1

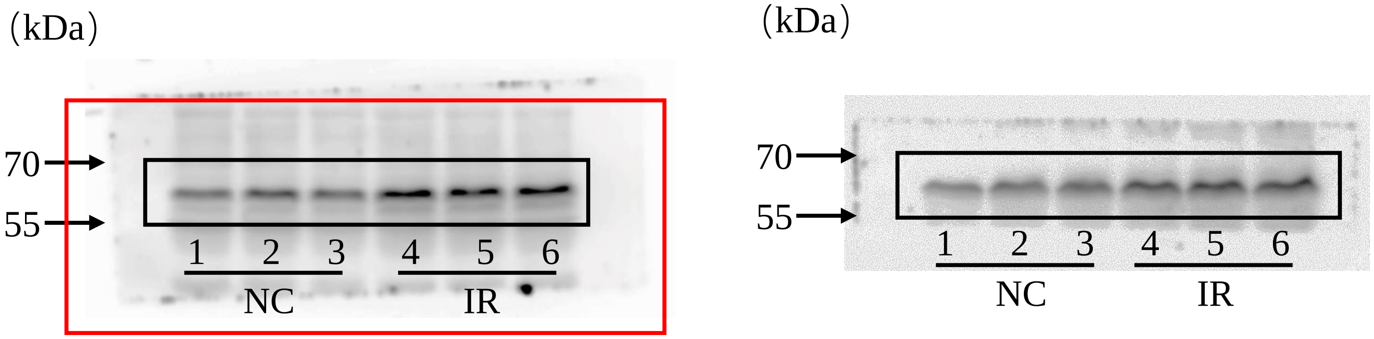

p62

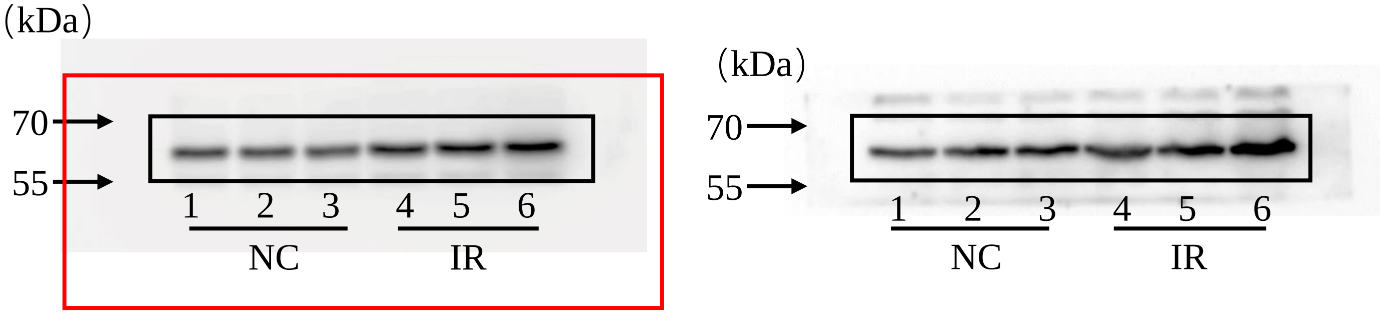

β-actin

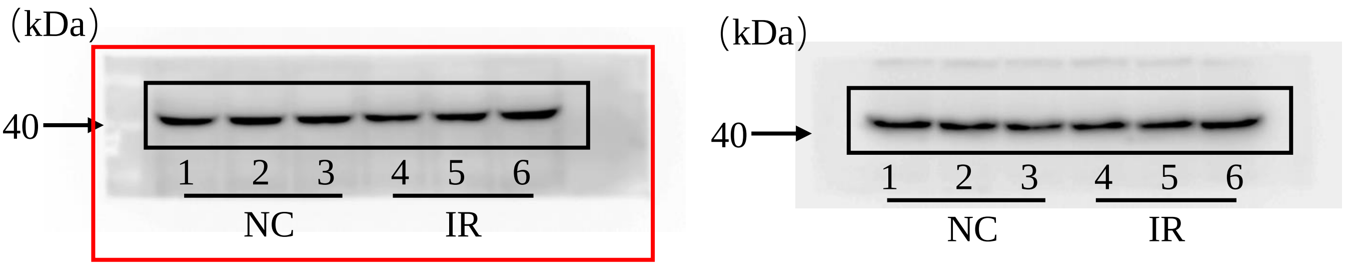

Figure 2E:

HIF-1α

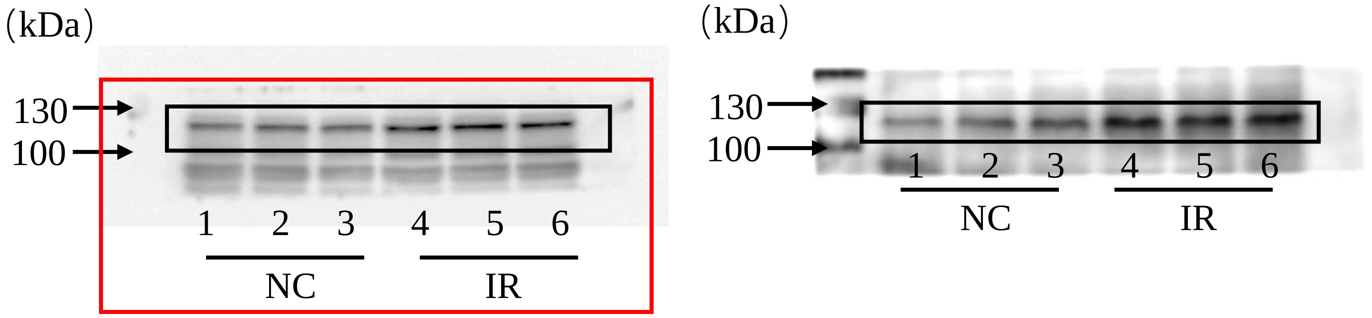

BNIP3

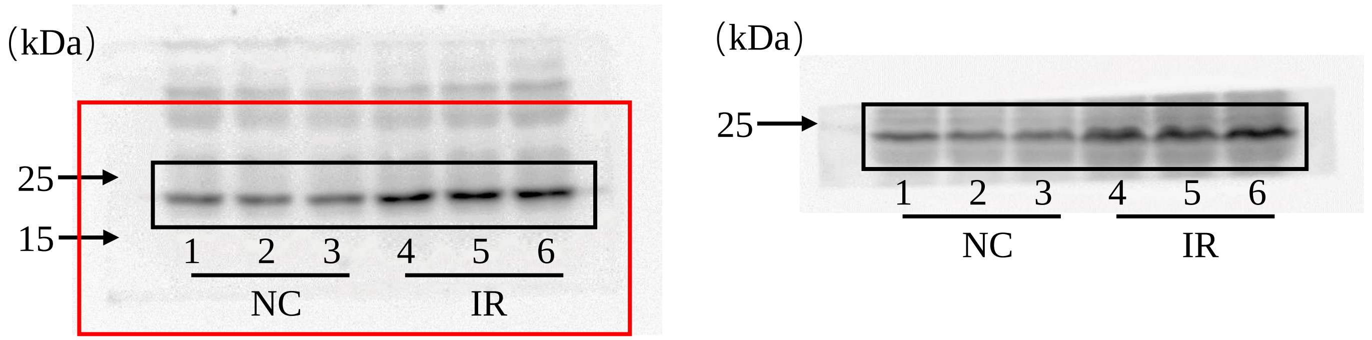

β-actin

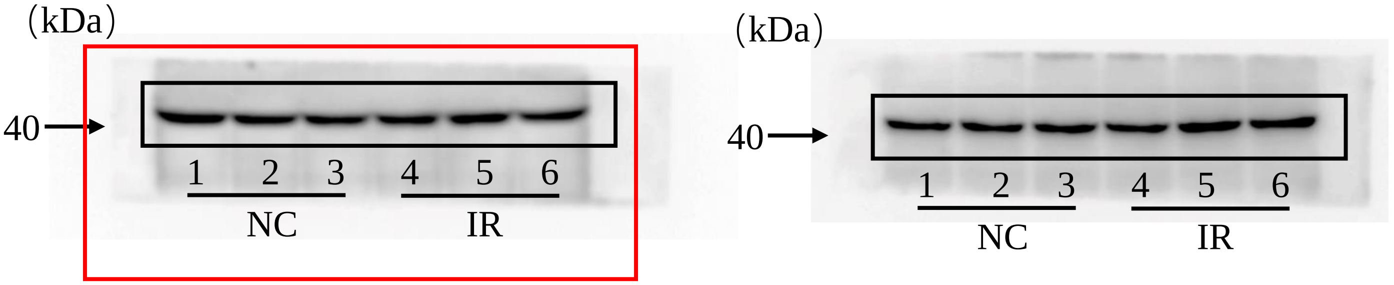

Figure 3F:

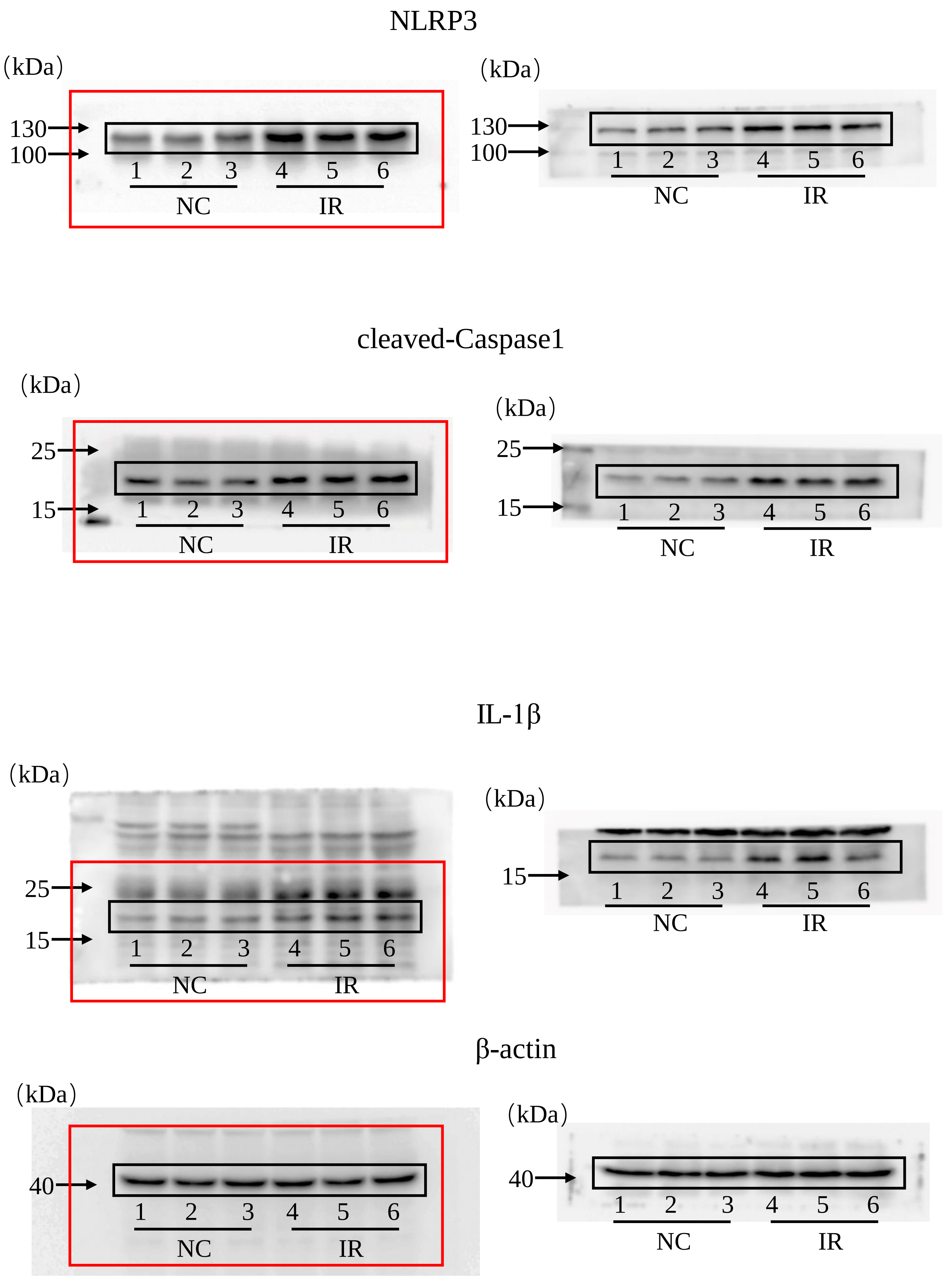

NLRP3
(kDa)
130
100
1 2 3 4 5 6
NC
IR
(kDa)
130
100
1 2 3 4 5 6
NC
IR
cleaved-Caspase1
(kDa)
25
15
1 2 3 4 5 6
NC
IR
(kDa)
25
15
1 2 3 4 5 6
NC
IR
IL-1β
(kDa)
25
15
1 2 3 4 5 6
NC
IR
(kDa)
15
1 2 3 4 5 6
NC
IR
β-actin
(kDa)
40
1 2 3 4 5 6
NC
IR
(kDa)
40
1 2 3 4 5 6
NC
IR

Figure 4B:

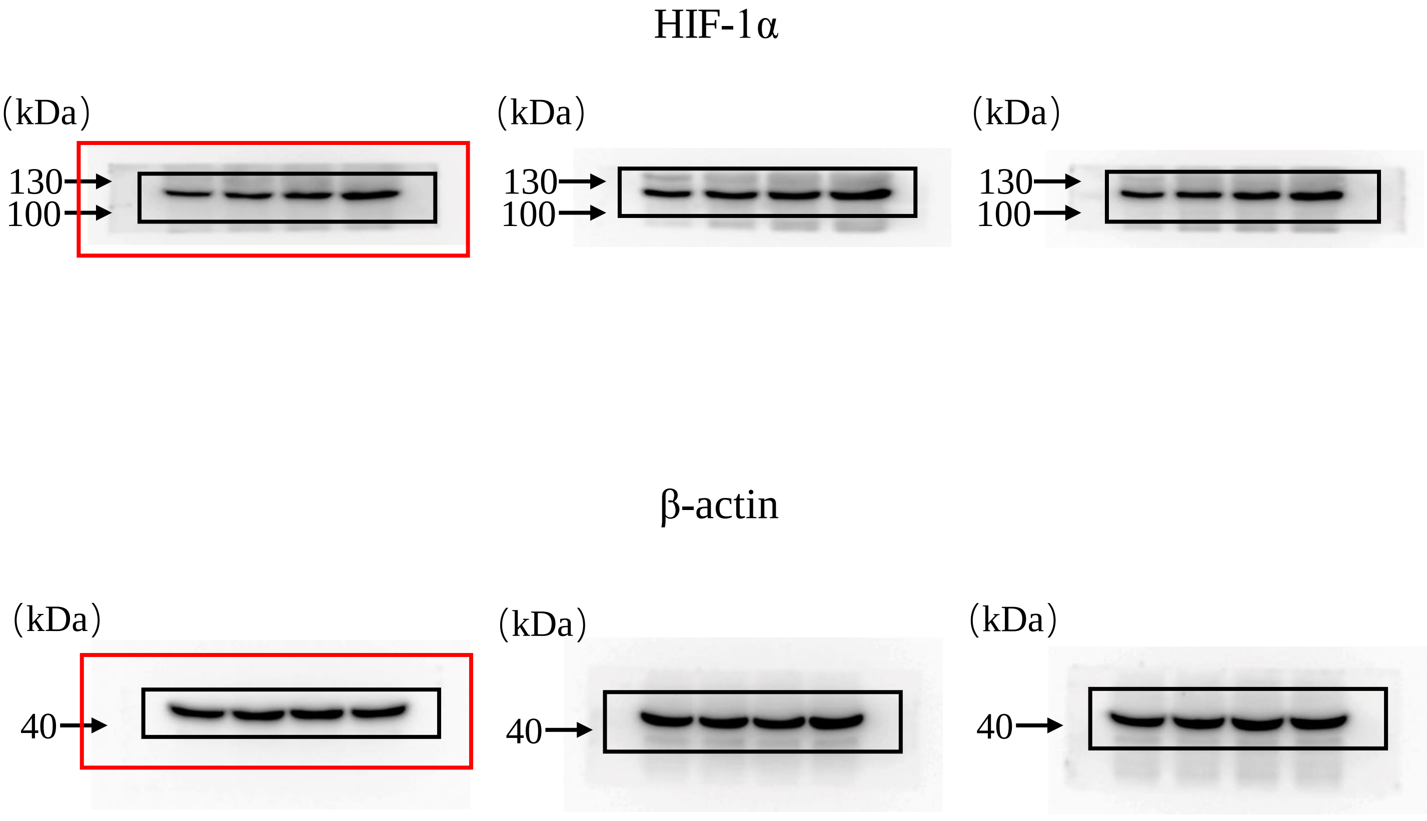

HIF-1α
(kDa)
130
100
(kDa)
130
100
(kDa)
130
100
β-actin
(kDa)
40
(kDa)
40
(kDa)
40

Figure 4E:

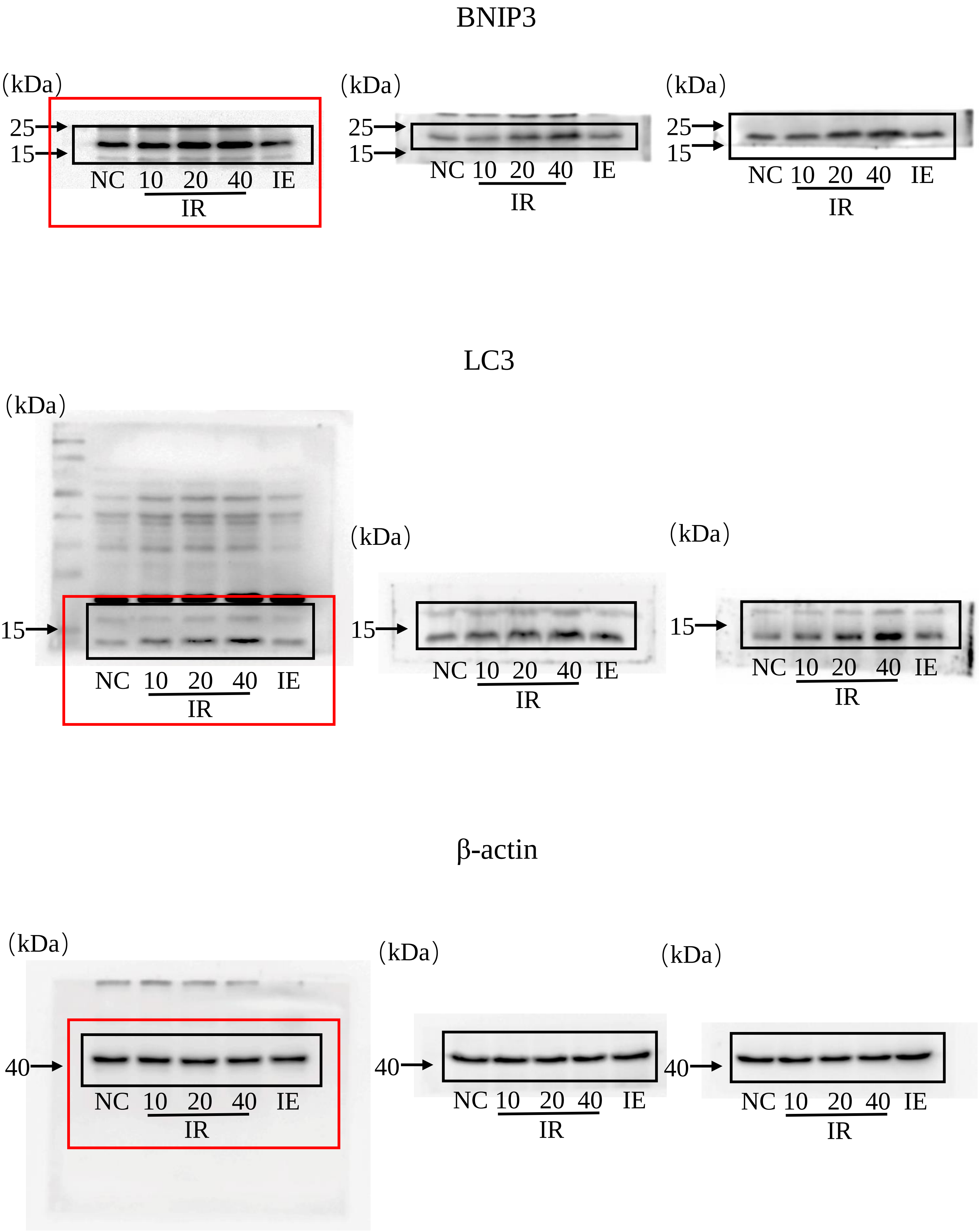
BNIP3
(kDa)
25
15
NC 10 20 40 IE
IR
LC3
(kDa)
15
NC 10 20 40 IE
IR
β-actin
(kDa)
40
NC 10 20 40 IE
IR

Figure 4E:

Beclin1

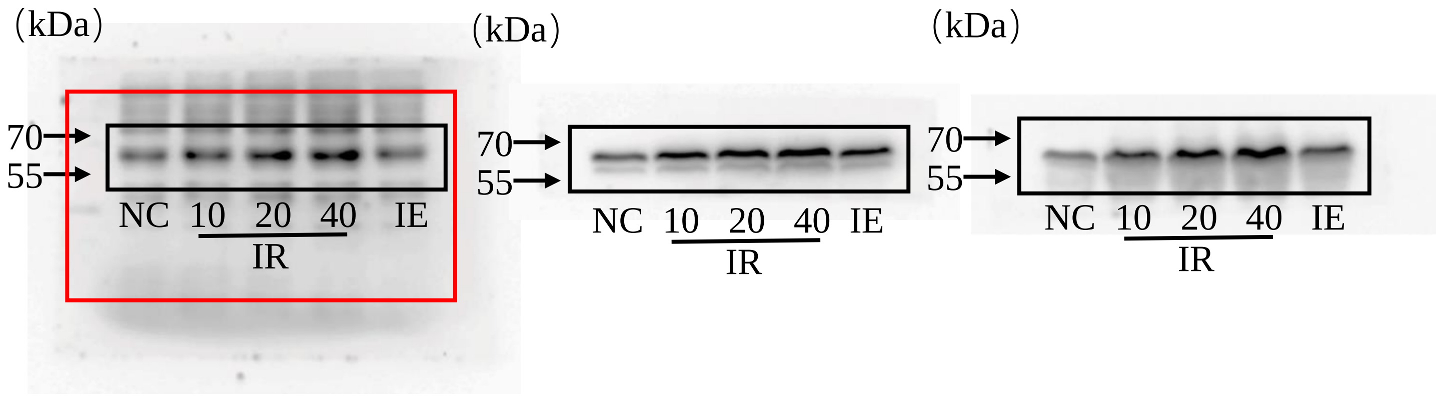

p62

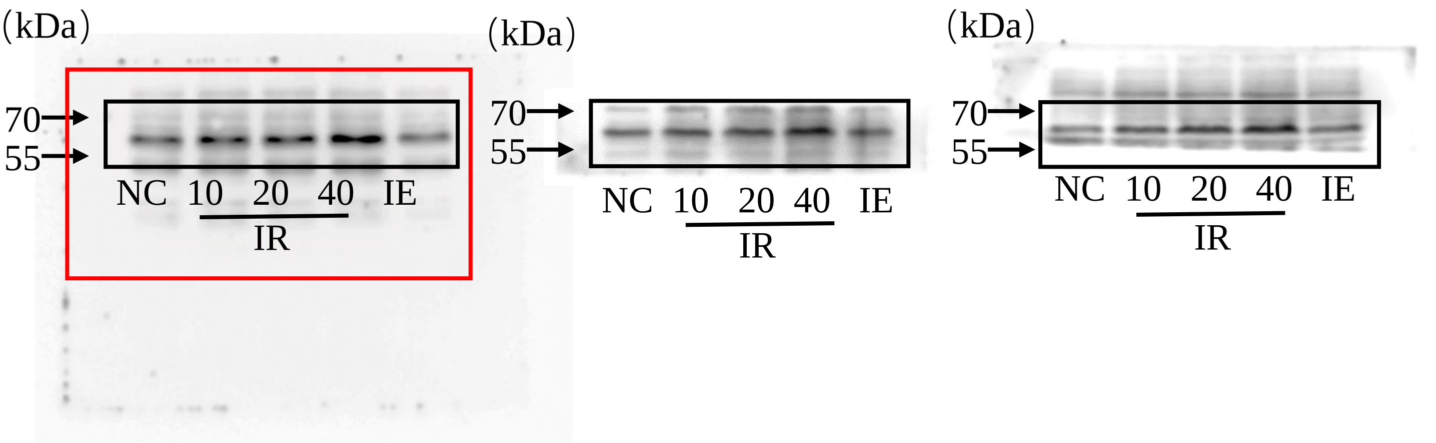

β-actin

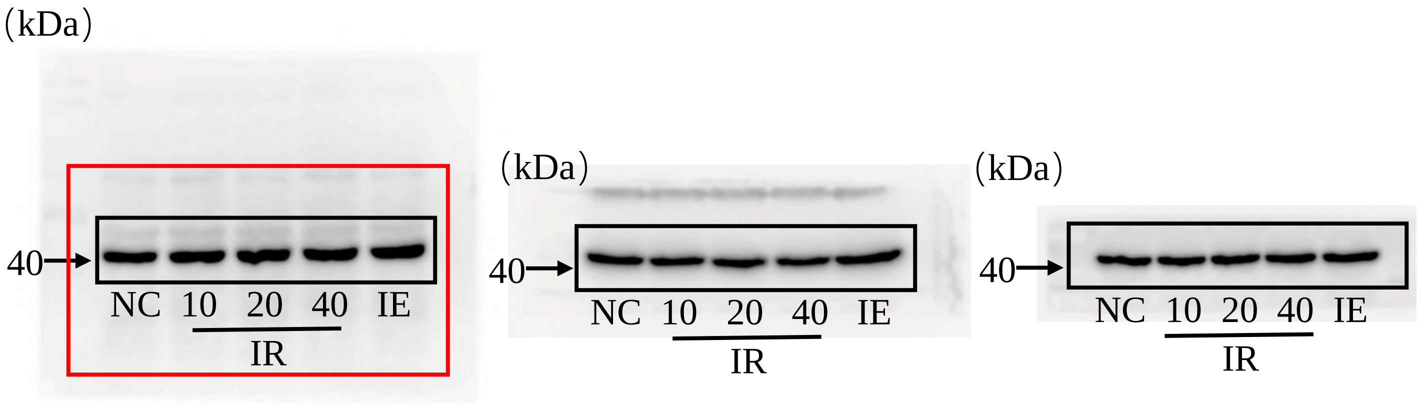

Figure 4H:

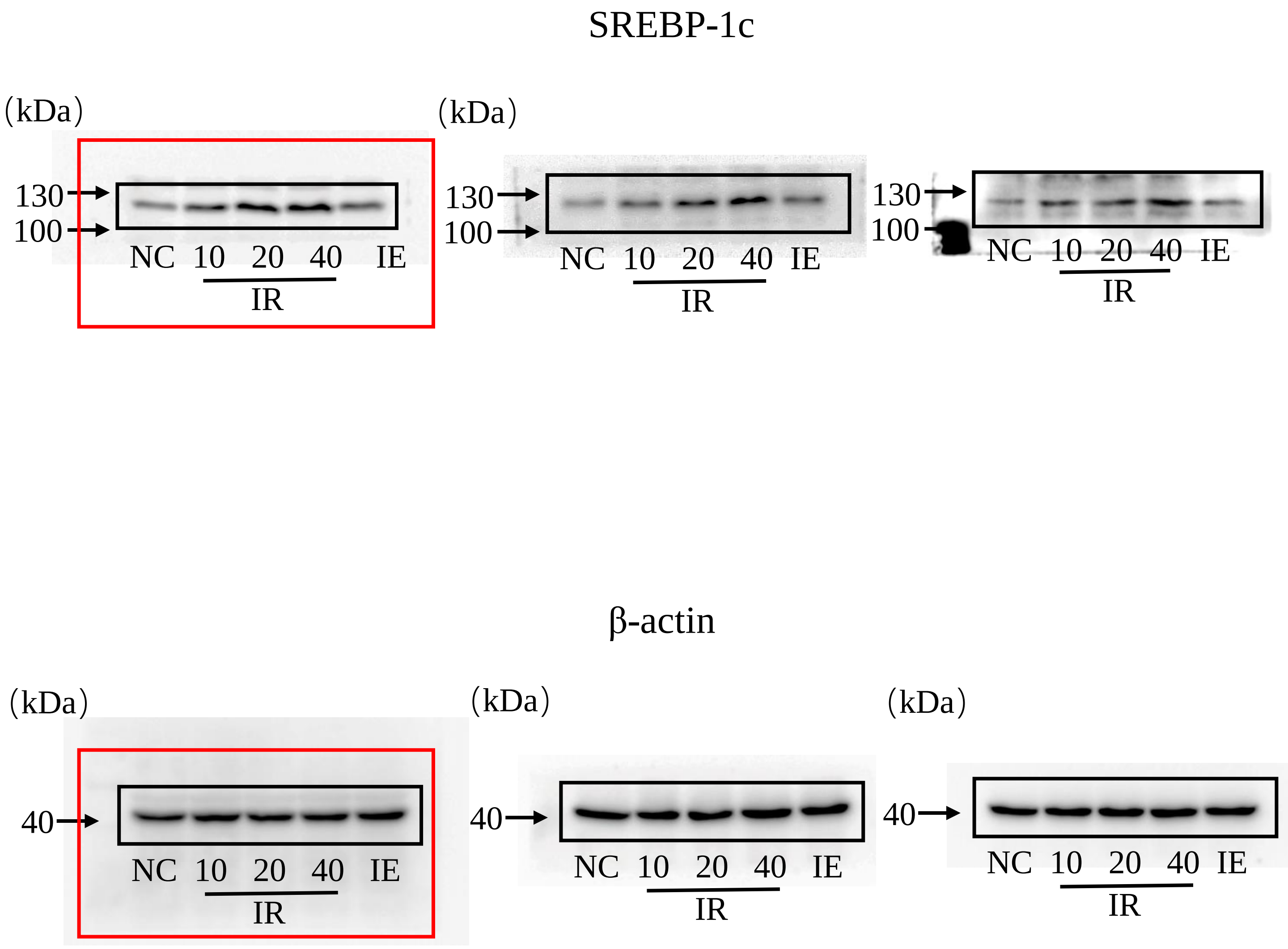

SREBP-1c
(kDa)
130
100
NC 10 20 40 IE
IR
β-actin
(kDa)
40
NC 10 20 40 IE
IR

Figure 5A:

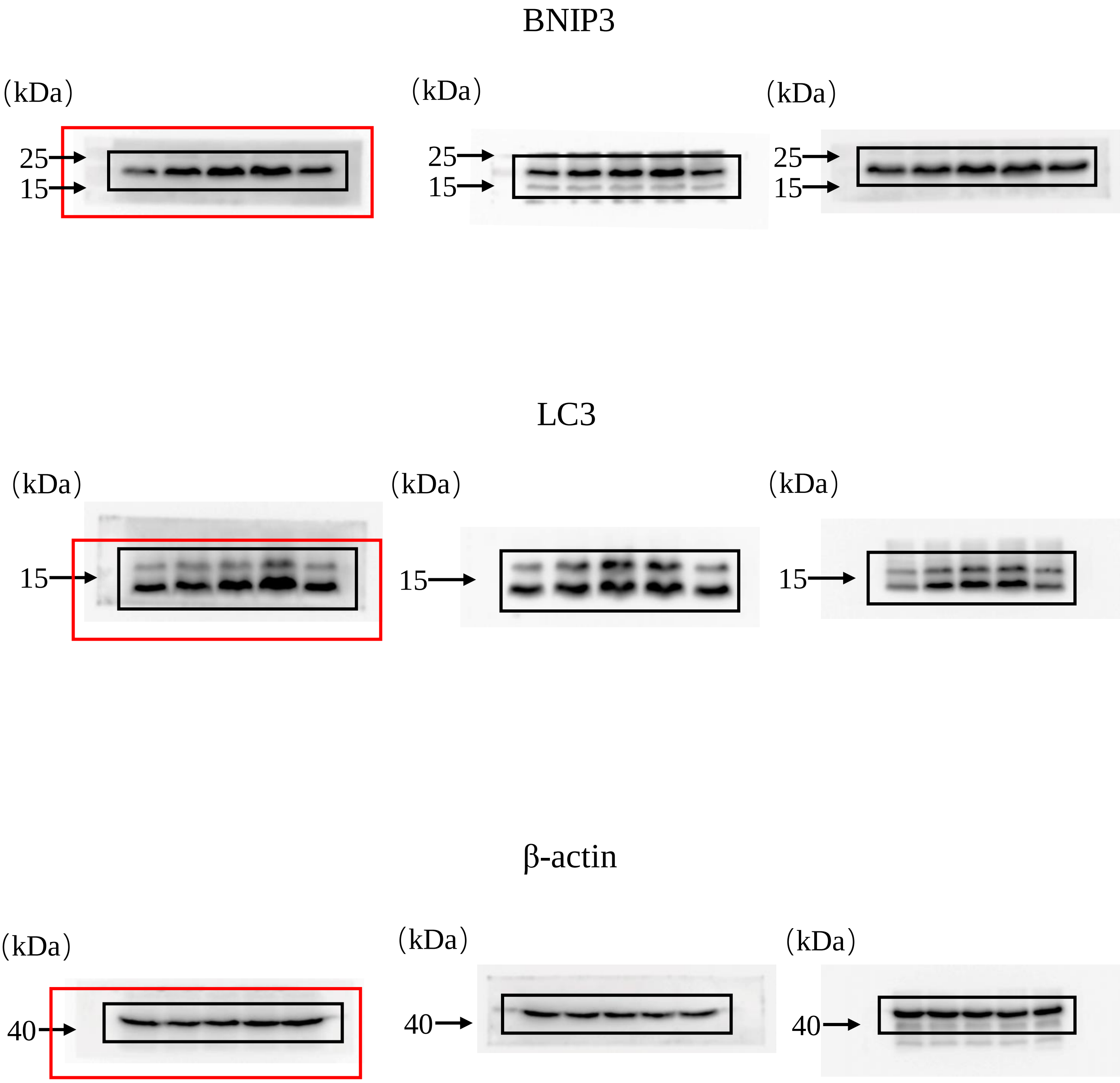
BNIP3
(kDa)
25
15
(kDa)
25
15
(kDa)
25
15
LC3
(kDa)
15
(kDa)
15
(kDa)
15
β-actin
(kDa)
40
(kDa)
40
(kDa)
40

Figure 5A:

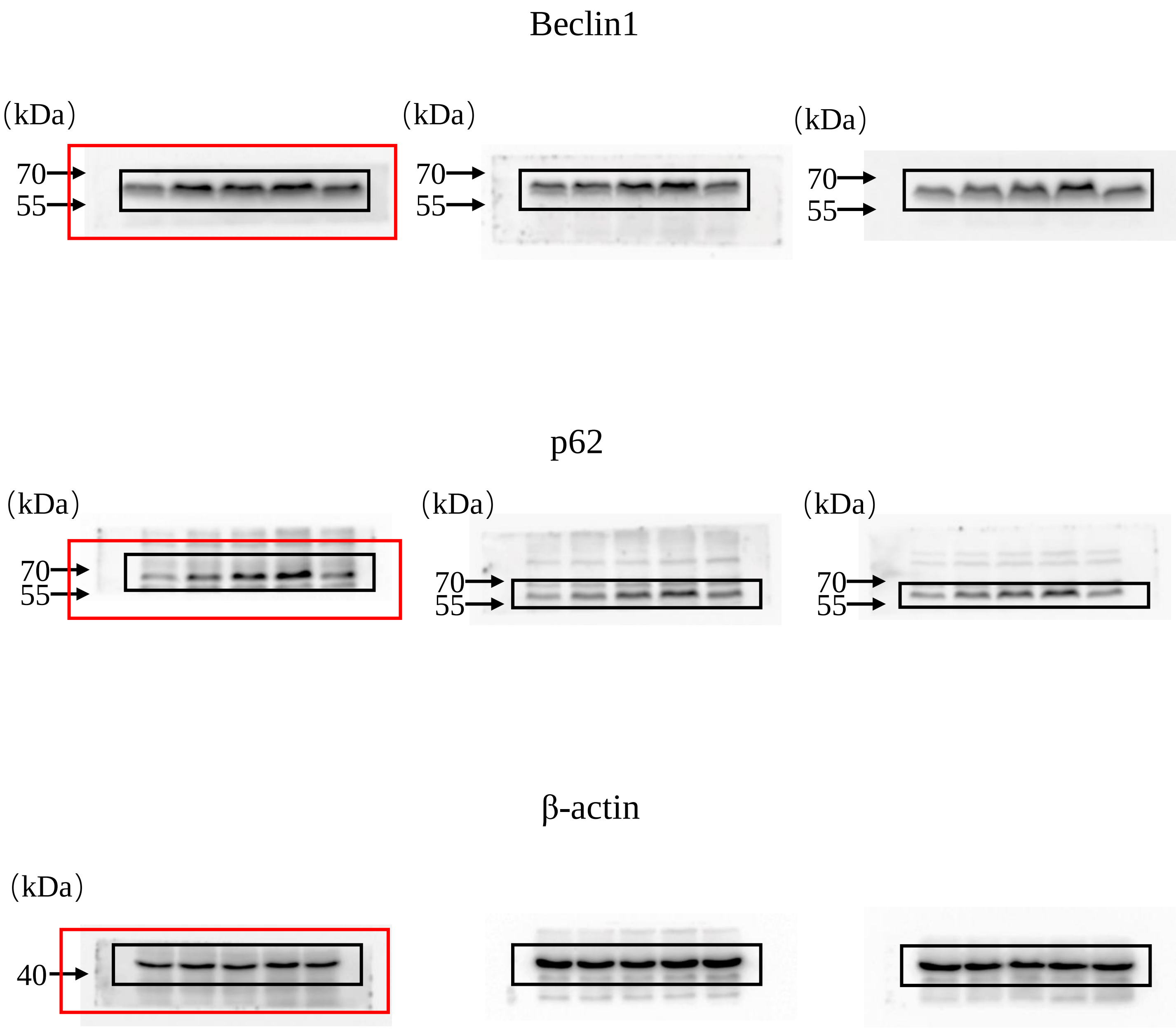
Beclin1
(kDa)
70
55
(kDa)
70
55
(kDa)
70
55
p62
(kDa)
70
55
(kDa)
70
55
(kDa)
70
55
β-actin
(kDa)
40

Figure 6A:

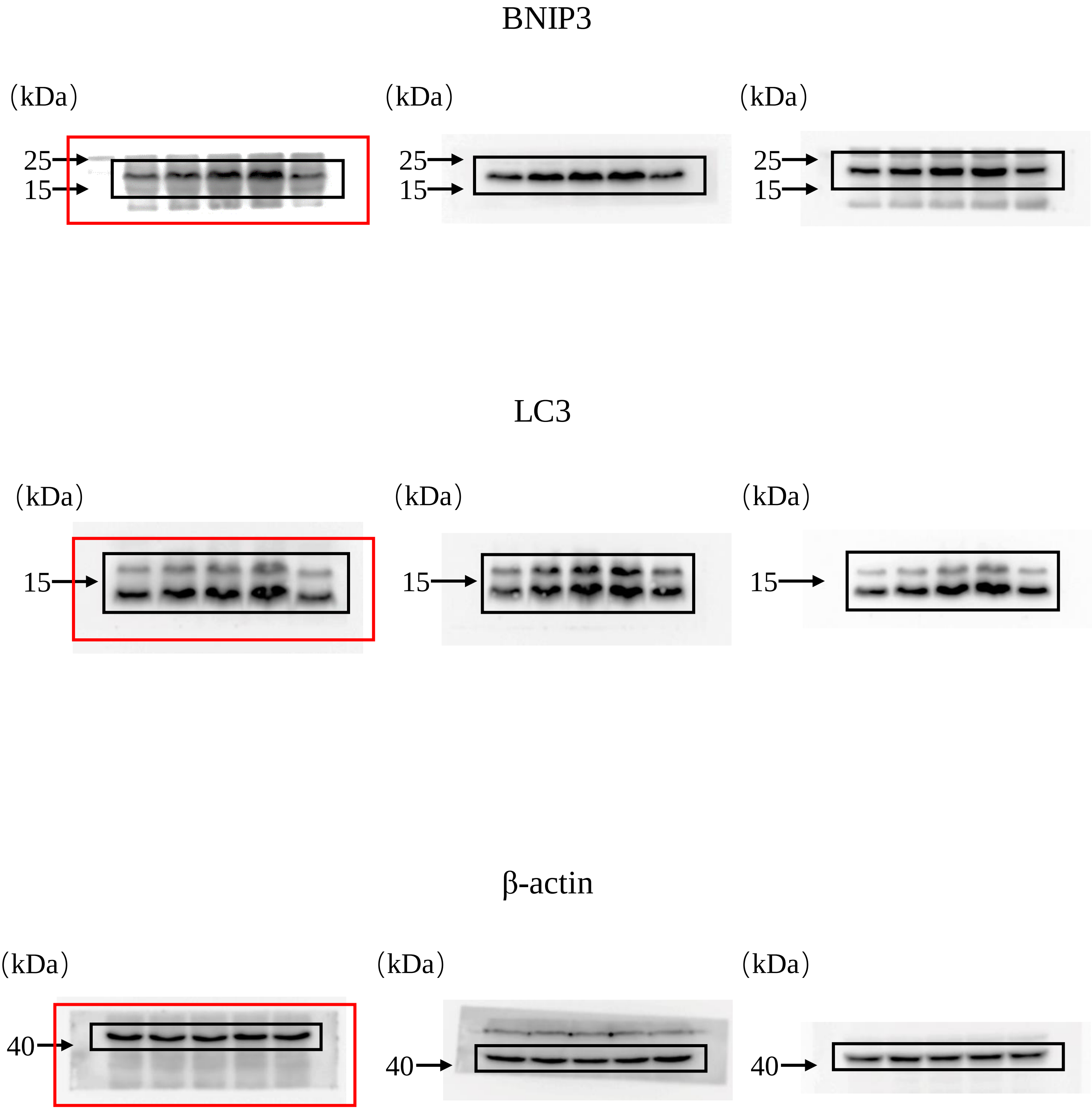
BNIP3
(kDa)
25
15
(kDa)
25
15
(kDa)
25
15
LC3
(kDa)
15
(kDa)
15
(kDa)
15
β-actin
(kDa)
40
(kDa)
40
(kDa)
40

Figure 6A:

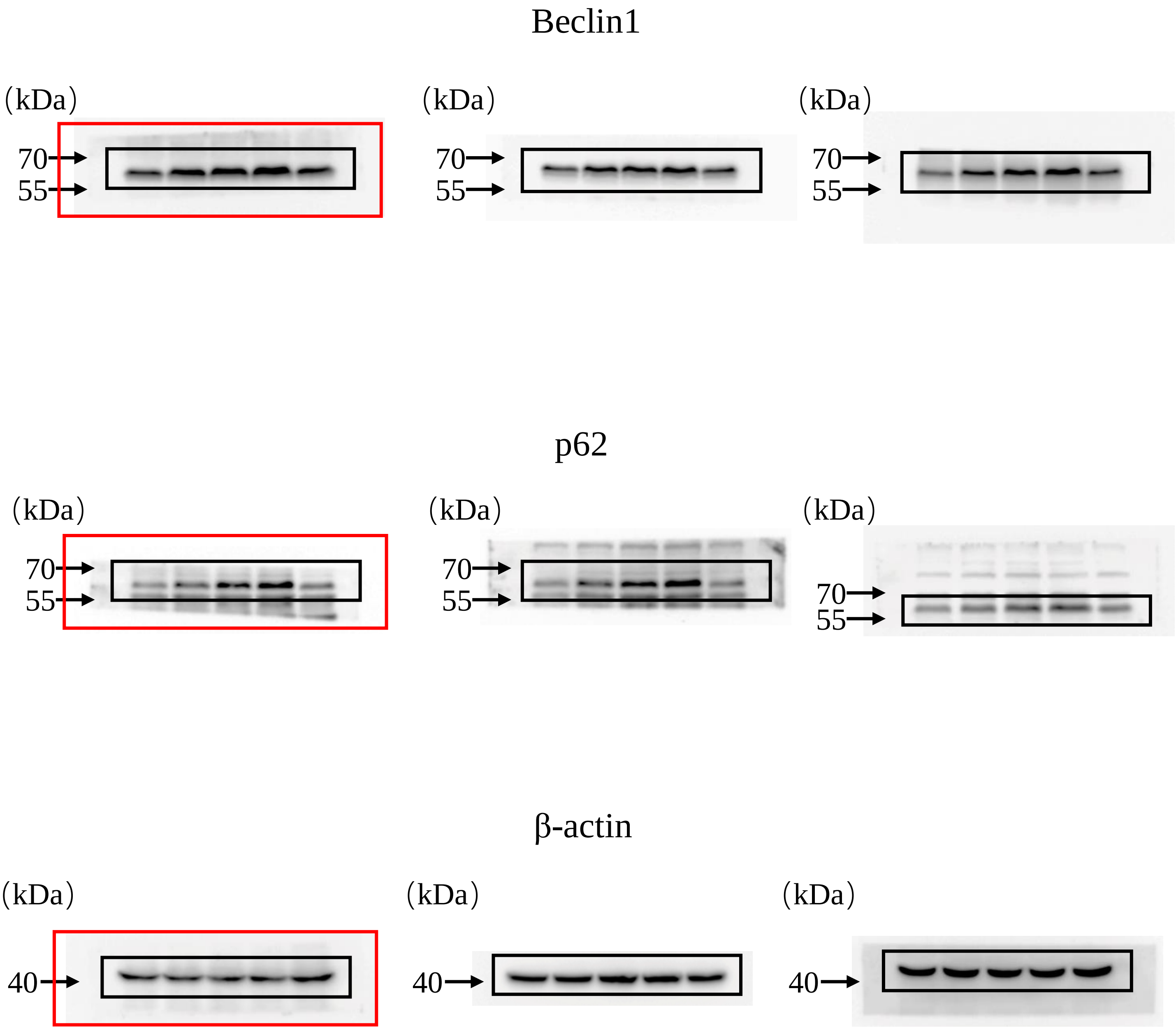
Beclin1
(kDa)
70
55
(kDa)
70
55
(kDa)
70
55
p62
(kDa)
70
55
(kDa)
70
55
(kDa)
70
55
β-actin
(kDa)
40
(kDa)
40
(kDa)
40

Figure 7A:

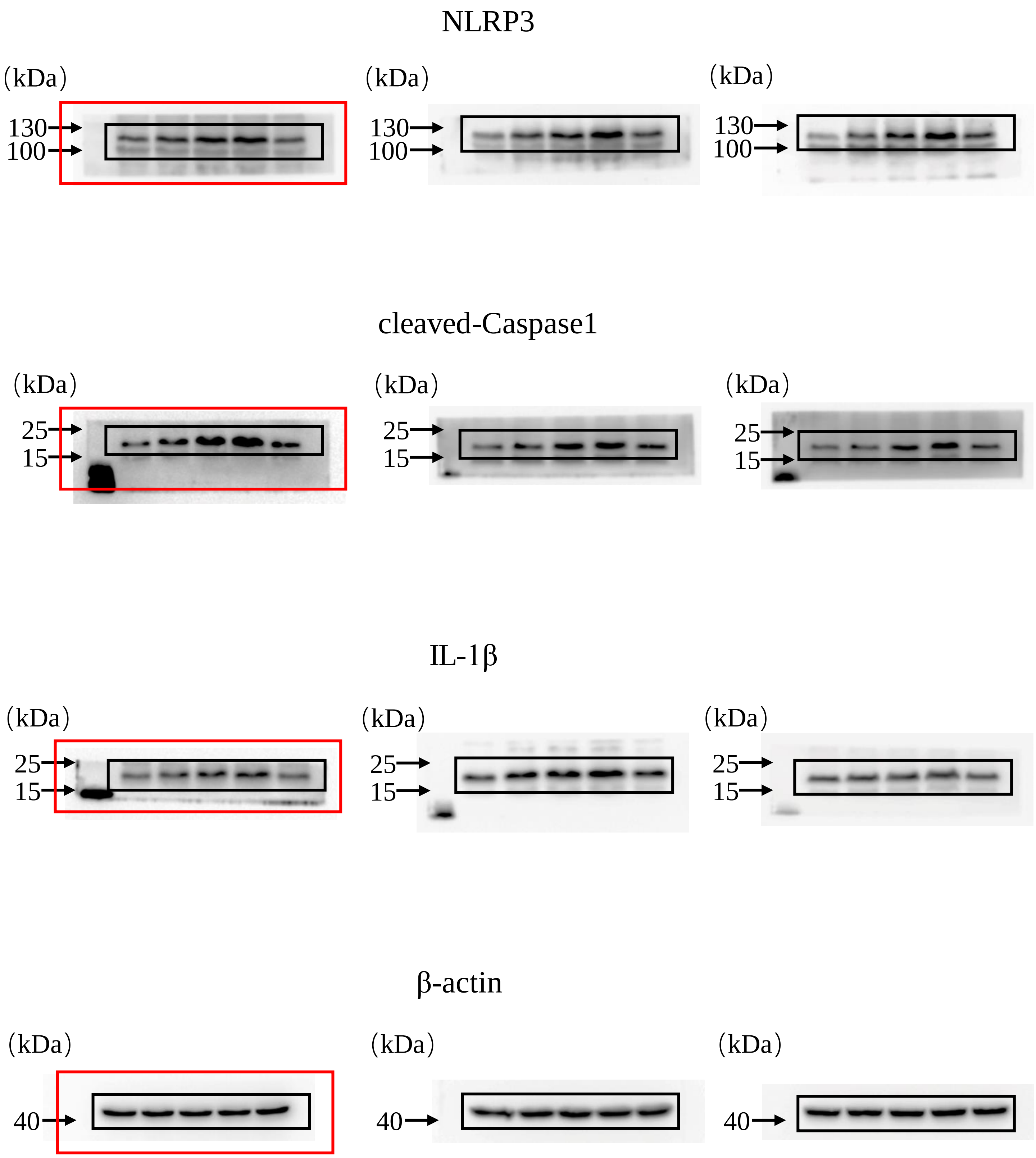
NLRP3
(kDa)
130
100
cleaved-Caspase1
(kDa)
25
15
IL-1β
(kDa)
25
15
β-actin
(kDa)
40

Figure 7D:

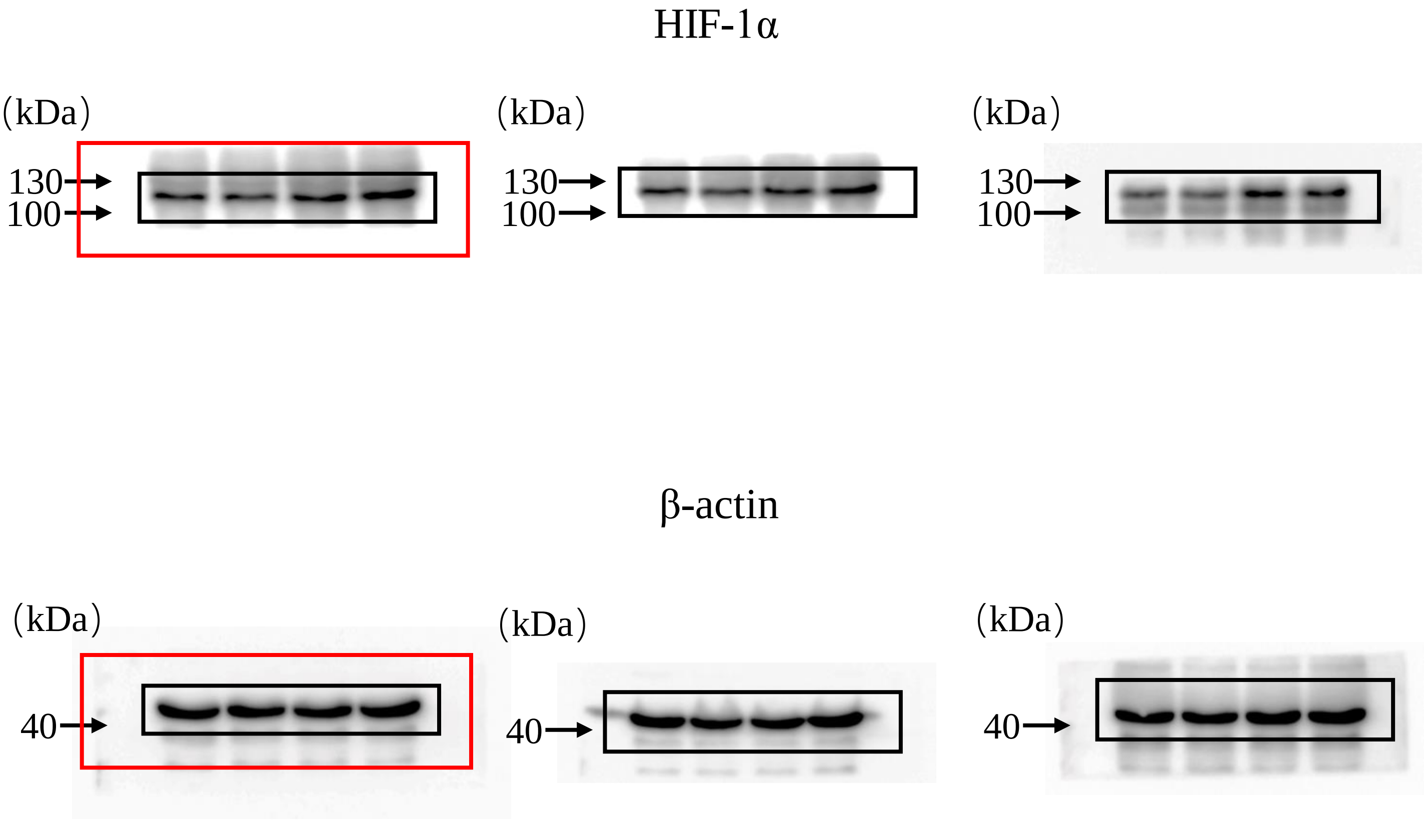
HIF-1α
(kDa)
130
100
(kDa)
130
100
(kDa)
130
100
β-actin
(kDa)
40
(kDa)
40
(kDa)
40

Figure 7D:

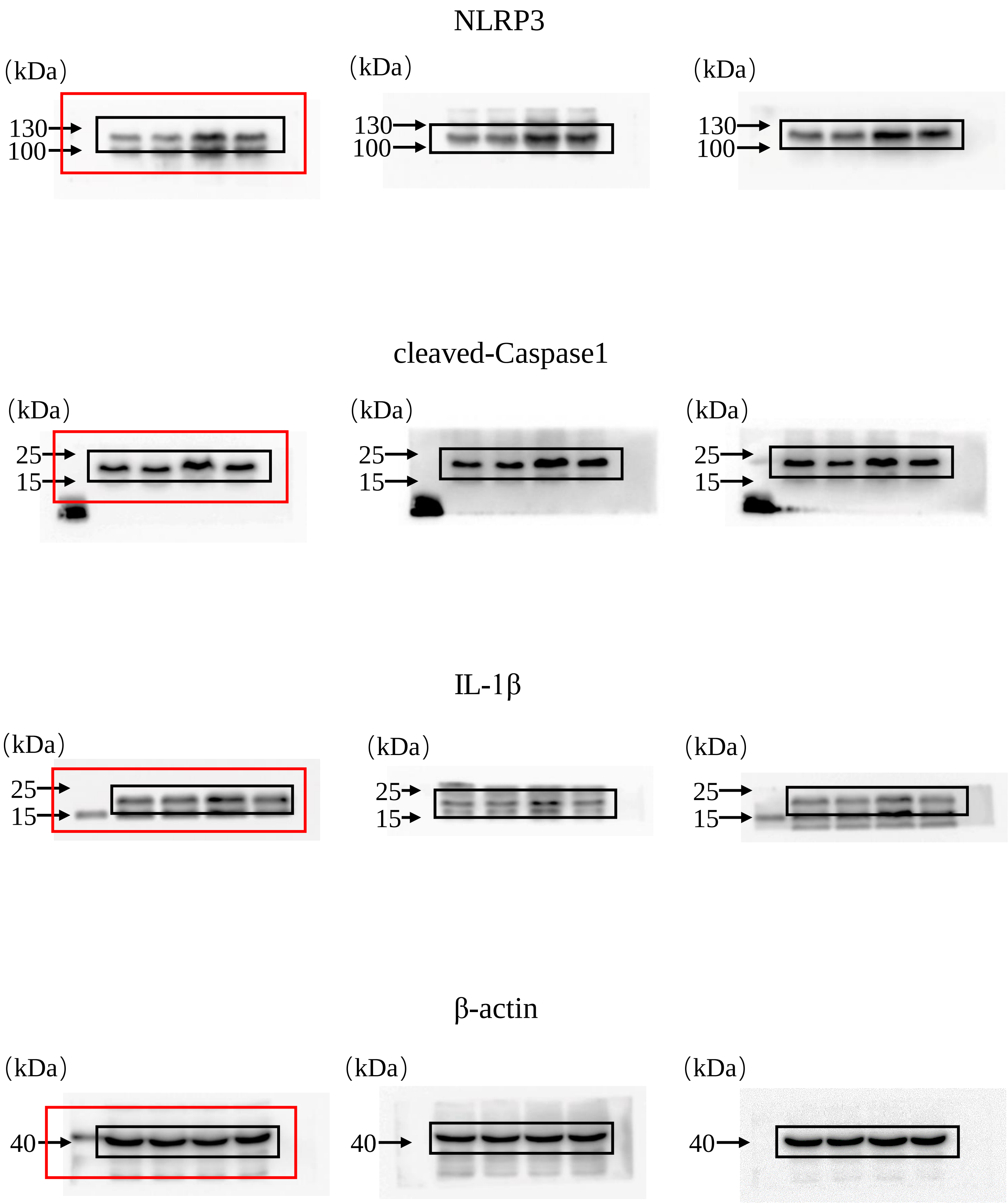
NLRP3
(kDa)
130
100
cleaved-Caspase1
(kDa)
25
15
IL-1β
(kDa)
25
15
β-actin
(kDa)
40

Figure S1:

HIF-1α

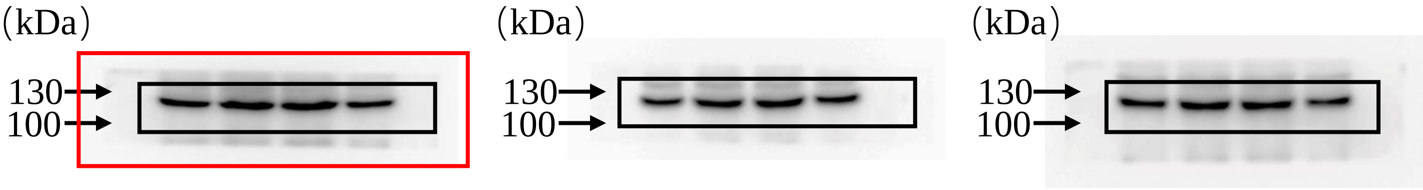

β-actin

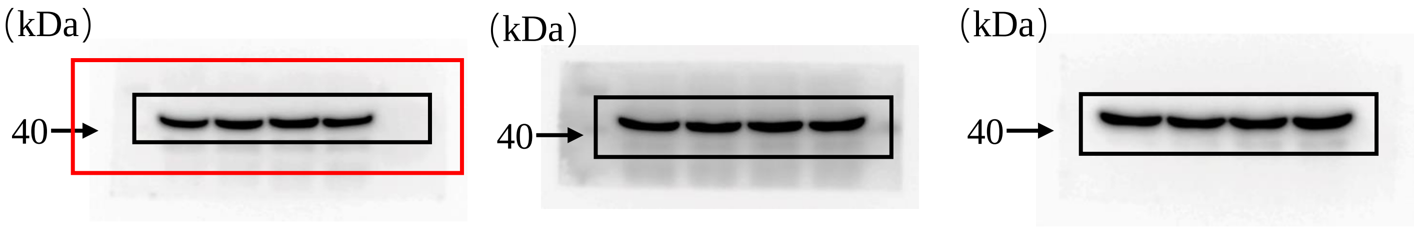

Supplement: Supplementary file 2 — Supplementary Information 2. [file 41598_2023_33848_MOESM2_ESM.pdf]
